# Metabolic Reprogramming of T Cells by Dual UCP2 and IL‐17 Blockade Enhances Immunity Against Pancreatic Cancer

**DOI:** 10.1002/advs.202513020

**Published:** 2026-01-04

**Authors:** Chuan‐Teng Liu, Chun‐Chieh Yeh, Tsai‐Chen Wu, Chia‐Hsin Lin, Yi‐Ting Kuo, Yoichiro Iwakura, Yuan‐Ji Day, Charles Drake, Vedran Radojcic, Ying‐Chyi Song, Heng‐Hsiung Wu, Hung‐Rong Yen

**Affiliations:** ^1^ Research Center for Traditional Chinese Medicine Department of Medical Research China Medical University Taichung Taiwan; ^2^ Chinese Medicine Research Center China Medical University Taichung Taiwan; ^3^ Department of Surgery Organ Transplantation Center China Medical University Hospital Taichung Taiwan; ^4^ School of Medicine College of Medicine China Medical University Taichung Taiwan; ^5^ Graduate Institute of Chinese Medicine School of Chinese Medicine College of Chinese Medicine China Medical University Taichung Taiwan; ^6^ Department of Chinese Medicine Dalin Tzu Chi Hospital Buddhist Tzu Chi Medical Foundation Chiayi Taiwan; ^7^ Research Institute For Biomedical Sciences Tokyo University of Science Tokyo Japan; ^8^ Department of Anesthesiology Tung's Taichung Metroharbor Hospital Taichung Taiwan; ^9^ Columbia Center For Translational Immunology Columbia University Irving Medical Center New York USA; ^10^ Department of Urology Columbia University Irving Medical Center New York USA; ^11^ Division of Hematology/Oncology Columbia University Irving Medical Center New York USA; ^12^ Division of Hematology & Hematologic Malignancies Department of Internal Medicine Huntsman Cancer Institute University of Utah Salt Lake City Utah USA; ^13^ Syndax Pharmaceuticals Waltham Massachusetts USA; ^14^ Graduate Institute of Integrated Medicine College of Chinese Medicine China Medical University Taichung Taiwan; ^15^ Research Center For Cancer Biology China Medical University Taichung Taiwan; ^16^ Drug Development Center China Medical University Taichung Taiwan; ^17^ The Ph.D. Program for Cancer Biology and Drug Discovery China Medical University and Academia Sinica Taichung Taiwan; ^18^ Division of Integration of Chinese and Western Medicine, Department of Chinese Medicine China Medical University Hospital Taichung Taiwan; ^19^ Office of Research and Development Asia University Taichung Taiwan

**Keywords:** cytotoxic T cell, immunotherapy, interleukin‐17, pancreatic cancer, uncoupling protein 2

## Abstract

Pancreatic ductal adenocarcinoma (PDAC) remains resistant to immunotherapy due to its immunosuppressive tumor microenvironment (TME) and impaired metabolic fitness of effector T cells. Here, we show that targeting UCP2 reprograms T‐cell metabolism, and that dual blockade with IL‐17 further enhance antitumor responses in PDAC. Pharmacologic UCP2 inhibition with genipin increases IFN‐γ production by CD8⁺ T cells through IL‐12R/STAT4/mTOR signaling and enhanced mitochondrial oxidative phosphorylation, promoting a T‐bet‐driven cytotoxic program. However, UCP2 inhibition alone does not suppress tumor growth. Accordingly, combination with IL‐17 depletion synergistically augments Tc1/Th1 responses, reduces myeloid‐derived suppressor cells (MDSCs), and improves survival across multiple PDAC models, including genetically engineered and orthotopic systems. CD8⁺ T‐cell depletion abrogates these effects. Moreover, UCP2 inhibition enhances IFN‐γ production in patient‐derived PBMCs and tumor‐infiltrating lymphocytes. These findings identify UCP2 as a metabolic checkpoint in cytotoxic T cells and support dual UCP2/IL‐17 blockade as a promising immunotherapeutic strategy for PDAC.

## Graphical abstract

This study demonstrates that dual UCP2/IL‐17 blockade reprograms T‐cell metabolism to overcome PDAC immunosuppression. Genipin‐mediated UCP2 inhibition enhances CD8⁺ T‐cell IFN‐γ via IL‐12R/STAT4/mTOR signaling and mitochondrial OXPHOS. Combined IL‐17 depletion amplifies Tc1/Th1 responses, reduces MDSCs, and prolongs survival in PDAC models, identifying UCP2 as a metabolic checkpoint and therapeutic target.

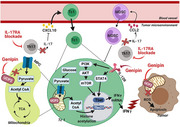

## 1 Introduction

Pancreatic ductal adenocarcinoma (PDAC) remains a largely incurable disease, primarily due to late diagnosis and the limited efficacy of systemic chemotherapies, which is partly attributed to the robust desmoplastic tumor microenvironment (TME) that promotes immunosuppression and chemoresistance [1]. Although cancer immunotherapy has emerged as a promising therapeutic avenue for PDAC, its effectiveness is severely restricted by the dense immunosuppressive TME [2]. Recent strategies that integrate immune effector activation with TME reprogramming have demonstrated improved antitumor efficacy in PDAC models [3].

Despite progress in immune checkpoint blockade, immunotherapy failure is still common, often driven by metabolic insufficiency in effector T cells [4]. Therefore, enhancing the metabolic fitness of immune effectors has become an attractive strategy to potentiate immunotherapy responses [4]. The uncoupling protein 2 (UCP2) is a mitochondrial membrane protein that regulates ATP synthesis and is broadly expressed in lymphohematopoietic tissues, where growing evidence indicates an immunomodulatory role. UCP2 deficiency in macrophages promotes pathogen clearance through metabolic reprogramming toward glycolysis and increased reactive oxygen species (ROS) production [5, 6], but exacerbates autoimmune inflammation by amplifying proinflammatory signals in disease models [7]. Beyond its role in immune regulation, tumors exploit UCP2 to protect against ROS and sustain tumorigenesis. Thus, targeting UCP2 may not only potentiate antitumor immunity but also sensitize tumor cells to cytotoxic attack.

Optimization of the TME to maintain cytotoxic T‐cell (Tc) activity and prevent cancer resistance to Tc‐mediated killing is essential for successful immunotherapy in PDAC. T helper 17 (Th17) and interleukin‐17 (IL‐17)‐producing cells infiltrate diverse malignancies and are frequently associated with poor prognosis [8]. In PDAC, elevated IL‑17⁺ T‐cell frequencies in the stroma correlate with advanced disease stage and shorter survival, while stromal Th17 accumulation promotes progression from PanIN to invasive PDAC [9]. In vivo, IL‐17 blockade enhances antitumor responses and prolongs survival in metastatic cancer models [10, 11]. These findings suggest that suppressing IL‐17 may synergize with UCP2 blockade to strengthen antitumor immunity in PDAC.

In this study, we evaluate the therapeutic potential of metabolic reprogramming and cytokine‐axis modulation through combined UCP2 and IL‐17 blockade in preclinical PDAC models and delineate mechanisms by which this approach enhances local and systemic antitumor immunity to restrain PDAC growth. We show that UCP2 inhibition with genipin, a small‐molecule inhibitor extracted from *Gardenia jasminoides* Ellis, augments antitumor immunity and that dual UCP2 and IL‐17 blockade synergistically suppresses tumorigenesis and prolongs survival in PDAC‐bearing mice. We further link these effects to metabolic reinforcement of T‐cell bioenergetics and mitigation of suppressive cues within the TME. Our work reveals a mechanistically grounded combinatorial immunotherapy strategy with promising efficacy in malignancies refractory to current immunotherapy interventions.

## 2 Results

### 2.1 UCP2 Blockade Promotes Type I Effector Polarization

**FIGURE 1:**
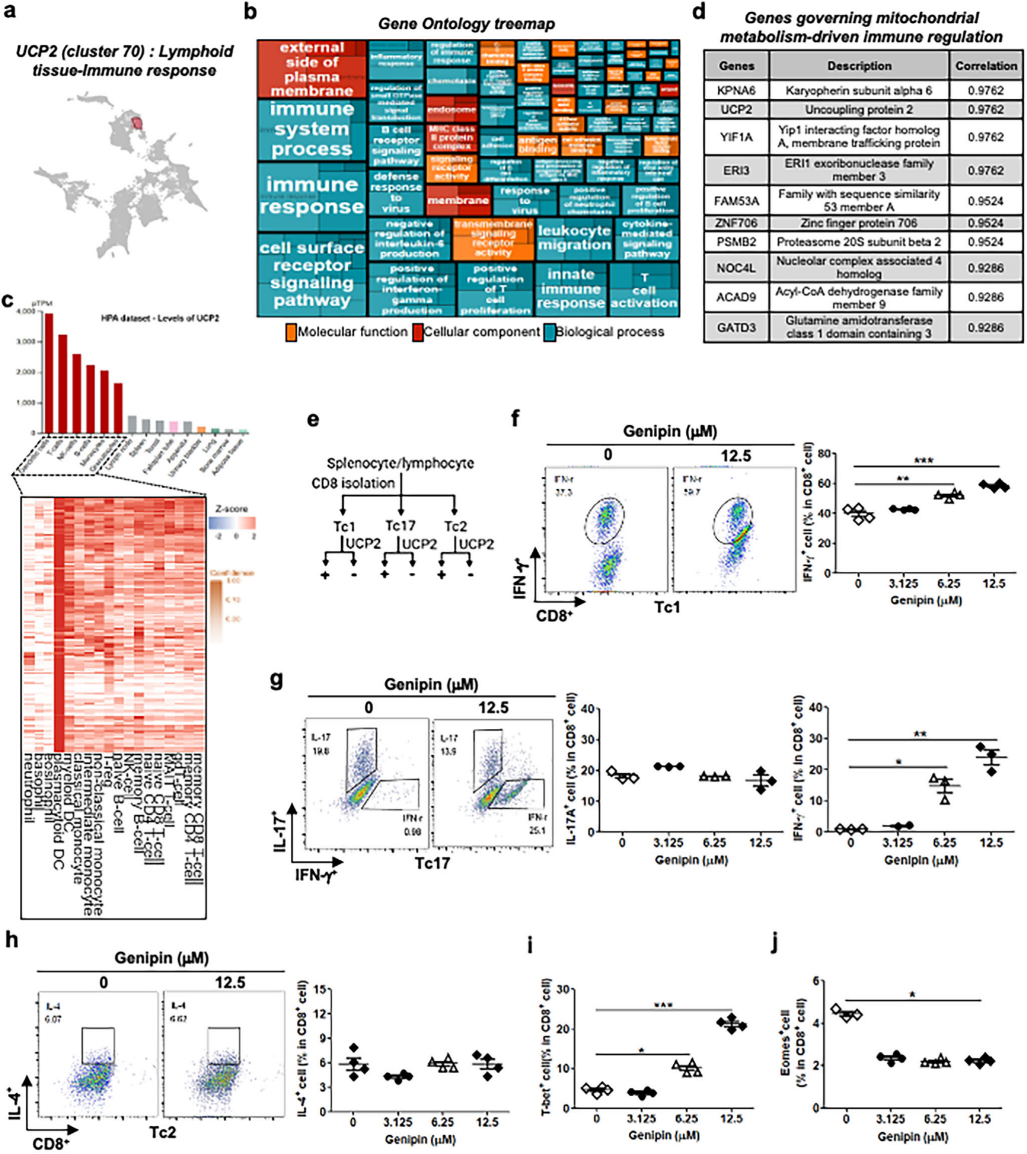
Targeting UCP2 enhances cytotoxic T‐cell polarization and antitumor immunity. (a–d) Gene ontology treemap, protein‐expression heatmap, and mitochondrial metabolism‐associated immune‐regulatory gene signatures based on UCP2 expression across human tissues. Transcript levels were normalized to transcripts per million protein‐coding genes (pTPM) using the Human Protein Atlas single‐cell transcriptomic dataset. (e) Experimental schema illustrating UCP2 blockade in polarized Tc subsets. (f) Representative flow‐cytometry plots and quantification of IFN‐γ production in genipin‐treated Tc1 cells (*n* = 4). (g) Representative flow‐cytometry plots and quantification of IFN‐γ and IL‐17 expression in genipin‐treated Tc17 cells (*n* = 3). (h) Representative flow‐cytometry plots and quantification of IL‐4 expression in genipin‐treated Tc2 cells (*n* = 4). (i,j) Intracellular cytokine staining (ICS) analysis of T‐bet (*n* = 4; i) and Eomes (*n* = 4; j) expression in IFN‐γ⁺CD8⁺ T cells following UCP2 inhibition in Tc1 cultures. Data represent mean ± SEM. ^*^ *p* < 0.05; ^**^ *p* < 0.01; ^***^ *p* < 0.001.

Single‐cell transcriptomic analysis reveals that UCP2 is included in cluster 70, which is associated with “Lymphoid tissue—Immune response” and linked to genes involved in mitochondrial metabolism‐driven immune regulation, underscoring its potential role in immune function (Figure 1a,b). UCP2 is expressed in multiple immune cell types, with dendritic cells showing the highest levels and T lymphocytes exhibiting relatively enriched expression (Figure 1c). As a key regulator of mitochondrial metabolism, UCP2 plays a pivotal role in immune cell function and metabolic adaptation (Figure 1d). Consistent with earlier observations that UCP2 inhibition enhances T‐cell activation—including antigen‐driven proliferation and TNF‐α and IL‐2 production in CD4⁺ T cells in autoimmune settings [12] —we examined the effect of UCP2 blockade with genipin on Tc and Th cell polarization (Figure 1e–j; Figure S1). UCP2 inhibition increased IFN‐γ expression in both Tc1 and Tc17 cells without affecting IL‐17 expression in Tc17 cells (Figure 1f,g), and did not alter IL‐4 expression in Tc2 cells (Figure 1h). UCP2 blockade elevated T‐bet expression but not Eomes, consistent with its role as a driver of type I effector differentiation (Figure 1i,j). Similarly, in CD4⁺ T cells, UCP2 inhibition increased IFN‐γ production in Th1 and Th17 cells without affecting IL‐17 expression (Figure S1b,c), and did not significantly alter Th2 or regulatory T‐cell (Treg) differentiation (Figure S1d,e). Together, these findings demonstrate that UCP2 suppression promotes antitumor immune potential by enhancing type I effector and helper phenotypes in CD8⁺ and CD4⁺ T cells.

### 2.2 UCP2 Blockade Augments IL‐12R/STAT4/mTOR Signaling and OXPHOS to Promote T‐bet‐Driven IFN‐γ Production in Tc1 Cells

**FIGURE 2:**
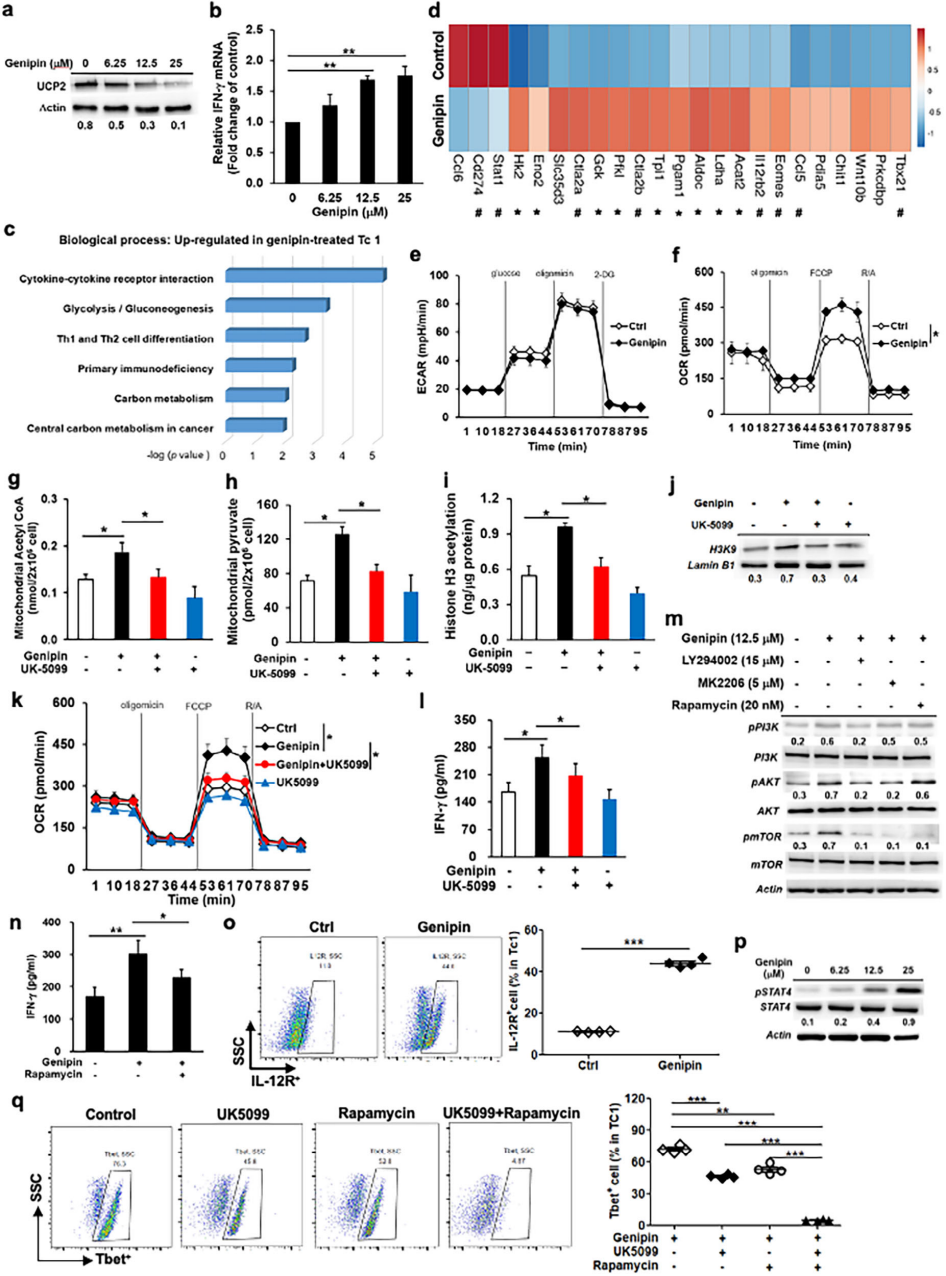
UCP2 inhibition enhances mitochondrial metabolism and IL‐12R/STAT4/mTOR signaling to drive T‐bet–dependent IFN‐γ production in Tc1 cells. (a) UCP2 protein expression in Tc1 cells treated with genipin for 12 h (representative immunoblot). (b) IFN‐γ mRNA expression following genipin treatment (*n* = 4). (c) Gene ontology biological processes (GOBPs) enriched in genipin‐treated Tc1 cells, highlighting pathways linked to T‐cell activation and metabolism. Enrichment scores were calculated by −log(P) using clusterProfiler. (d) Representative genes significantly upregulated by UCP2 inhibition associated with T‐cell activation (#) and metabolic regulation (*). (e,f) Bioenergetic profiling of genipin‐treated Tc1 cells: ECAR (*n* = 5; e) and OCR (*n* = 5; f) measured using a Seahorse XF24 analyzer. (g–l) Impact of mitochondrial pyruvate transport on genipin‐induced metabolic and functional changes. Mitochondrial acetyl‐CoA (*n* = 4; g), mitochondrial pyruvate (*n* = 4; h), histone H3 acetylation (*n* = 4; i), H3K9 acetylation (j), OCR (*n* = 5; k), and IFN‐γ production (*n* = 4; l) were quantified with or without UK5099 (40 µm). (m) Effects of PI3K/AKT/mTOR pathway inhibitors (LY294002, MK‐2206, rapamycin) on genipin‐induced PI3K, AKT, and mTOR phosphorylation (representative immunoblot). (n) Rapamycin‐mediated suppression of IFN‐γ expression in genipin‐treated Tc1 cells (*n* = 4). (o) Flow‐cytometry analysis of IL‐12R expression in genipin‐treated Tc1 cells (*n* = 4). (p) STAT4 phosphorylation following genipin stimulation for 12 h (representative immunoblot). (q) Effects of UK5099 and rapamycin on T‐bet expression in genipin‐treated Tc1 cells (representative flow‐cytometry plots; *n* = 4). Data represent mean ± SEM. ^*^ *p* < 0.05; ^**^ *p* < 0.01.

After verifying UCP2 and IFN‐γ expression in genipin‐treated Tc1 cells, we proceeded to assess their transcriptional profile upon UCP2 inhibition (Figure 2a,b). RNA‐seq analysis identified 1495 differentially expressed genes between genipin‐treated and DMSO‐treated Tc1 cells (Table S1). UCP2 blockade correlated positively with genes involved in IFN‐γ signaling, leukocyte activation, and bioenergetic metabolism (Figure 2c). Genipin increased the expression of glycolysis‐associated genes, including Hk2, Gck, Pfkl, Aldoc, Pgam1, Eno2, and Ldha (Figure 2d), indicating enhanced glycolytic programming and pyruvate generation in Tc1 cells. In parallel, genipin upregulated genes related to T‐cell activation and migration, including Tbx21, Ccl5, Eomes, Il12rb2, and Ctla2 (Figure 2d). A transient divergence between Eomes mRNA and protein expression was observed following UCP2 inhibition, reflecting distinct transcriptional and post‐transcriptional kinetics. Eomes transcripts increased at early activation phases but declined as T‐bet expression became dominant, consistent with prior evidence that sustained T‐bet upregulation suppresses Eomes to stabilize effector T‐cell differentiation [13]. Despite transcriptional induction of glycolytic genes, UCP2 inhibition did not increase ECAR, whereas it enhanced oxidative phosphorylation (OCR) in Tc1 cells (Figure 2e,f). Consistent with preferential mitochondrial carbon utilization, genipin elevated mitochondrial acetyl‐CoA (Figure 2g) and pyruvate levels (Figure 2h), accompanied by increased histone H3 acetylation (H3K9Ac), a modification associated with IFN‐γ locus accessibility (Figure 2i,j). These genipin‐driven metabolic effects were reversed by UK5099, a specific mitochondrial pyruvate carrier (MPC) inhibitor (Figure 2g–l), demonstrating that UCP2 blockade redirects glycolytic carbon toward mitochondrial oxidation rather than lactate production. This transcript–flux dissociation aligns with previous reports in activated T cells, and the MPC dependency is consistent with mitochondrial pyruvate utilization supporting effector function [14, 15]. To evaluate the T‐cell–intrinsic role of UCP2 in Tc1 programming, we analyzed CD8⁺ T cells from CD4Cre × UCP2^f/f^ mice. UCP2 deficiency enhanced Tc1 effector function, reflected by increased IFN‐γ production and T‐bet expression. Loss of UCP2 also augmented mitochondrial metabolism, including higher OCR, mitochondrial acetyl‐CoA, and mitochondrial pyruvate levels. Notably, all enhanced effector and metabolic features were reversed by the MPC inhibitor UK5099 (Figure S2a–e). Together, these results indicate that UCP2 limits Tc1 differentiation by restraining mitochondrial pyruvate utilization, and its inhibition enhances IFN‐γ production through increased oxidative metabolism, acetyl‐CoA accumulation, and epigenetic remodeling of the IFN‐γ locus.

To further investigate how UCP2 blockade influences IL‐12/IL‐12 receptor (IL‐12R), mTOR, PI3K, and STAT4 signaling—pathways essential for T‐bet‐dependent type I effector programming—we examined activation of these signaling nodes in Tc1 cells. Genipin increased mTOR and upstream PI3K and AKT phosphorylation. Pharmacologic inhibition of individual components using LY294002, MK‐2206, or rapamycin attenuated genipin‐induced PI3K, AKT, and mTOR activation (Figure 2m). Rapamycin significantly reduced IFN‐γ expression in genipin‐treated Tc1 cells (Figure 2n). Consistent with Il12rb2 upregulation in the RNA‐seq dataset, UCP2 blockade increased IL‐12R expression (Figure 2o) and enhanced STAT4 activation (Figure 2p). Both rapamycin and UK5099 prevented genipin‐induced T‐bet upregulation, with an additive inhibitory effect when combined (Figure 2q). Taken together, these findings demonstrate that UCP2 blockade enhances Tc1 metabolic and effector fitness in an IL‐12/STAT4/mTOR‐dependent manner.

### 2.3 UCP2 Inhibition Suppresses Tumorigenesis and Metastasis in PDAC Cells

In addition to its immunostimulatory effects, we investigated tumor‐intrinsic consequences of UCP2 inhibition in PDAC cells in vitro. Genipin was previously reported to induce apoptosis in PDAC cells through ROS accumulation [16]. Consistent with this, genipin reduced Pan18 cell viability in a dose‐dependent manner (Figure S3a) and significantly impaired tumorigenic and metastatic features, including soft‐agar colony formation and invasive capacity (Figure S3b,c). UCP2 inhibition further triggered apoptosis, lipid peroxidation, and increases in ROS and nitric oxide, accompanied by reduced GPx expression in Pan18 cells (Figure S3d–i). Metabolically, genipin shifted cellular bioenergetics from protumorigenic glycolysis toward antitumor oxidative phosphorylation (Figure S3j,k). To assess whether these antitumor effects extend across PDAC subtypes, we examined multiple human PDAC cell lines. Genipin reduced cell viability in Su.86.86, MiaPaCa 2, and Panc1 cells (Figure S4a), and similarly increased ROS levels while suppressing colony formation and invasion in Panc‐1 cells (Figure S4b–d). Together, these results demonstrate that UCP2 inhibition directly restricts PDAC tumorigenesis and metastatic potential by rewiring tumor‐cell metabolism and inducing oxidative stress, in addition to its immunostimulatory actions on cytotoxic T cells.

### 2.4 UCP2 Inhibition Alone is Insufficient to Suppress Tumor Growth in PDAC Models

**FIGURE 3:**
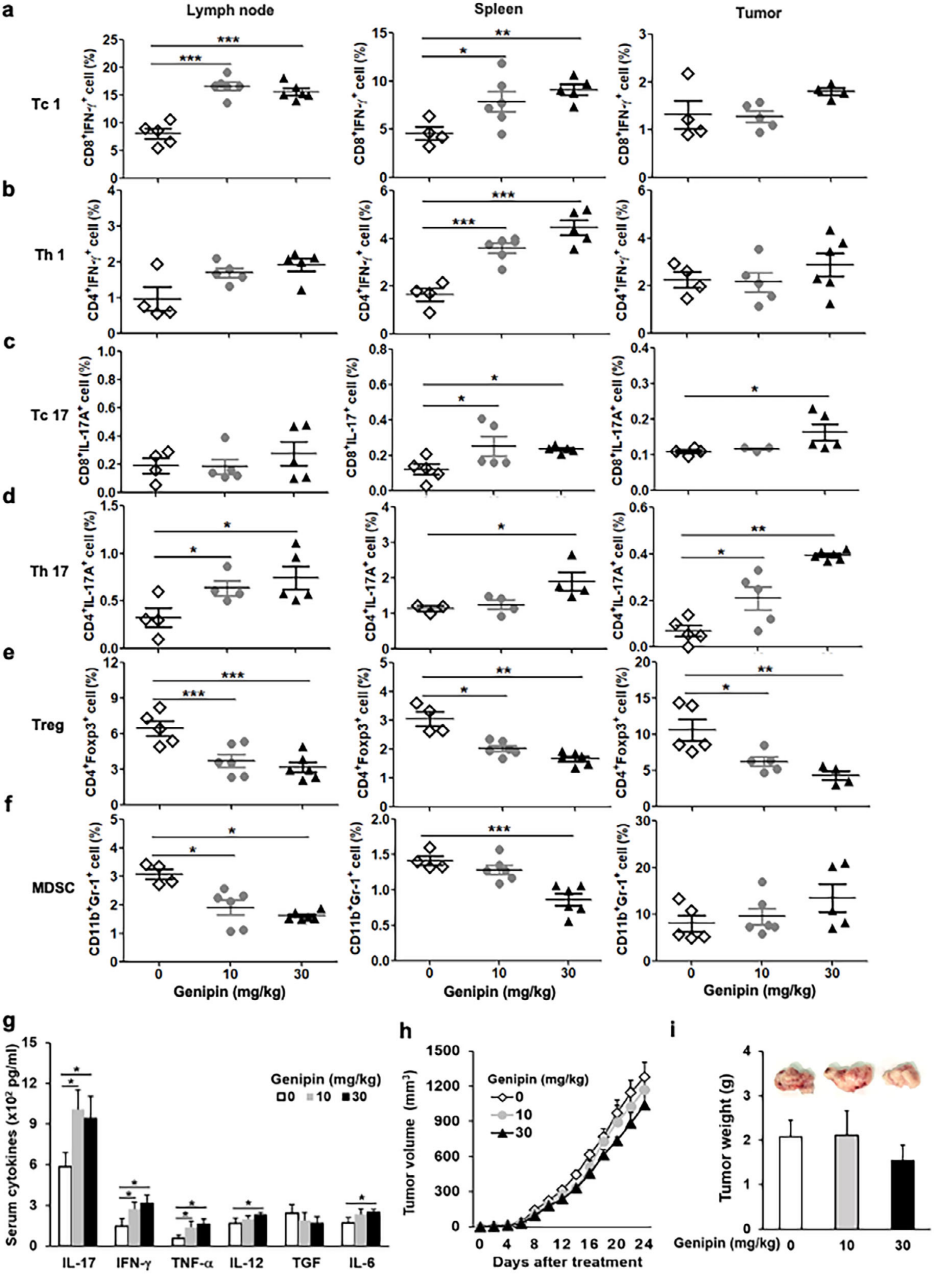
UCP2 inhibition enhances systemic antitumor immunity but does not impede tumor progression in a PDAC model. Wild‐type (WT) mice were implanted subcutaneously with Pan18 cells (*n* = 4). Genipin (10 or 30 mg/kg, i.p., every two days) was administered for 3 weeks. (a–g) Immune profiling in draining lymph nodes, spleen, and tumors of genipin‐treated PDAC‐bearing mice (*n* = 4). Flow cytometry was used to quantify Tc1 (a), Th1 (b), Tc17 (c), Th17 (d), Treg (e), and MDSC (f) populations. (g) Serum cytokines, including IL‐17, IFN‐γ, TNF‐α, IL‐12, TGF‐β, and IL‐6, were measured at study endpoint. (h) Tumor volume was monitored every two days, and (i) tumor weight was measured at sacrifice. Data are presented as mean ± SEM. ^*^ *p* < 0.05; ^**^ *p* < 0.01; ^***^ *p* < 0.001.

Given the immunostimulatory effects of genipin in vitro, we evaluated its antitumor efficacy in vivo using an ectopic mouse PDAC model. Immune profiling of spleen, tumor‐draining lymph nodes, and tumor tissues revealed that genipin treatment increased Tc1, Th1, Tc17, and Th17 populations while reducing Tregs and MDSCs, indicating enhanced effector T‐cell activity and attenuation of suppressive immune subsets (Figure 3a–f). Consistently, serum levels of IL‐17, IFN‐γ, TNF‐α, IL‐12, and IL‐6 were elevated in genipin‐treated tumor‐bearing mice (Figure 3g). Despite these favorable systemic immune changes, UCP2 inhibition alone did not reduce tumor burden in vivo (Figure 3h,i). This dissociation suggests that PDAC tumors retain immune‐evasion mechanisms that limit intratumoral recruitment or functionality of genipin‐induced Tc1 and Th1 cells. Notably, genipin also increased Th17 frequencies, which may counterbalance antitumor immunity, consistent with prior evidence that Th17 responses support PDAC progression and immune escape [11]. These observations prompted us to test whether targeting IL‐17 could relieve Th17‐mediated immunosuppression and enable UCP2‐driven cytotoxic T‐cell responses to exert effective tumor control.

### 2.5 Combined UCP2 and IL‐17 Blockade Suppresses Tumor Growth in Tumor‐Bearing Mice

To determine whether dual targeting of UCP2 and IL‐17 improves antitumor efficacy, we first examined IL‐17‐deficient (IL‐17^−/−^) mice. Interleukin‐17 depletion alone modestly inhibited tumor growth (Figure S5a,b) and was associated with increased serum IL‐12 and enhanced tumoral Tc1 infiltration, accompanied by reduced MDSCs (Figure S5c–g). Consistently, IL‐17 deficiency elevated CXCL10 and decreased CCL2, chemokines that respectively promote effector T‐cell recruitment and suppress myeloid infiltration into the TME (Figure S5h–j).

**FIGURE 4:**
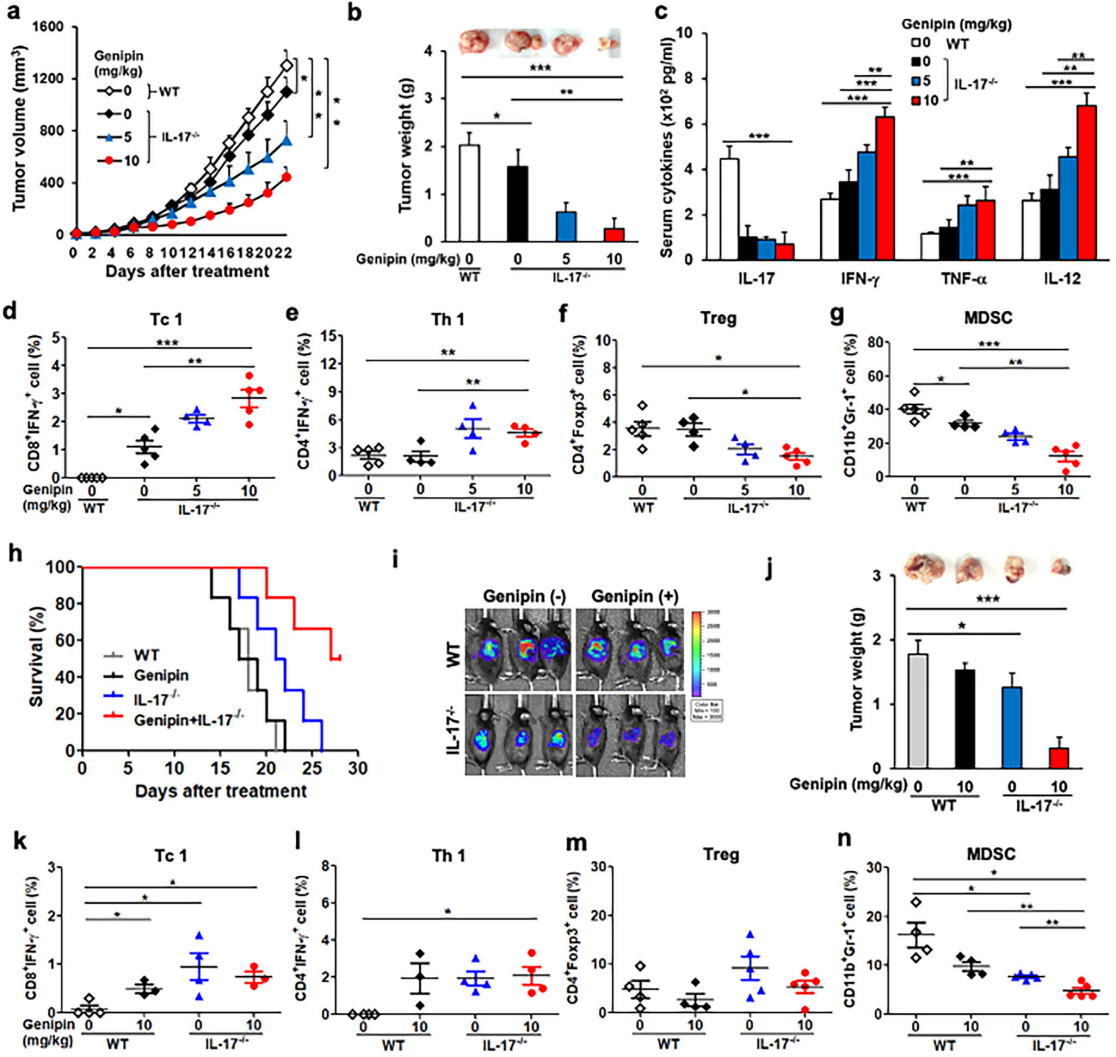
Dual UCP2 and IL‐17 blockade suppresses tumor growth and enhances antitumor immunity in PDAC models. IL‐17 knockout mice were implanted with Pan18 cells and treated with genipin (5 or 10 mg/kg, i.p., every two days) for 3 weeks (*n* = 4–5). (a) Tumor volume was monitored every two days. (b) Tumor weight was measured at study endpoint. (c–g) Immune profiling in PDAC‐bearing IL‐17^−/−^ mice following genipin treatment. Serum cytokines (IL‐17, IFN‐γ, TNF‐α, IL‐12) were quantified (c). Tumoral Tc1 (d), Th1 (e), Treg (f), and MDSC (g) populations were assessed by flow cytometry. For orthotopic modeling, WT and IL‐17^−/−^ mice were implanted with Pan18 cells and treated with genipin (10 mg/kg, i.p., every two days) for 3 weeks. (h) Overall survival (*n* = 6). (i) In vivo bioluminescence tumor monitoring (*n* = 5). (j) Tumor weight at necropsy (*n* = 5). (k–n) Flow‐cytometric analysis of intratumoral Tc1 (k), Th1 (l), Treg (m), and MDSC (n) populations in WT and IL‐17−/− mice treated with genipin (*n* = 4–5). Data are shown as mean ± SEM. ^*^ *p* < 0.05; ^**^ *p* < 0.01; ^***^ *p* < 0.001.

We next evaluated combinatorial inhibition of UCP2 and IL‐17 across multiple PDAC models. Dual blockade produced a synergistic reduction in tumor burden (Figure 4a,b). In PDAC‐bearing IL‐17^−/−^ mice, genipin further increased systemic IFN‐γ, IL‐12, and TNF‐α in a dose‐dependent manner (Figure 4c) and amplified intratumoral Tc1 and Th1 responses while reducing Tregs and MDSCs (Figure 4d–g). UCP2 inhibition did not further affect IL‐17‐dependent modulation of CXCL10 and CCL2 (Figure S6a,b). Immunohistochemistry confirmed enhanced lipid peroxidation and increased CD3⁺ T‐cell infiltration following dual blockade (Figure S6c,d). Importantly, genipin alone or in combination with IL‐17 blockade did not induce body‐weight loss or hepatic/renal toxicity (Figure S6e,f).

In an orthotopic PDAC model, dual blockade again yielded a robust antitumor effect and survival advantage compared with single‐agent therapy (Figure 4h–j), associated with elevated Tc1/Th1 infiltration and reduced tumoral MDSCs (Figure 4k–n).

**FIGURE 5:**
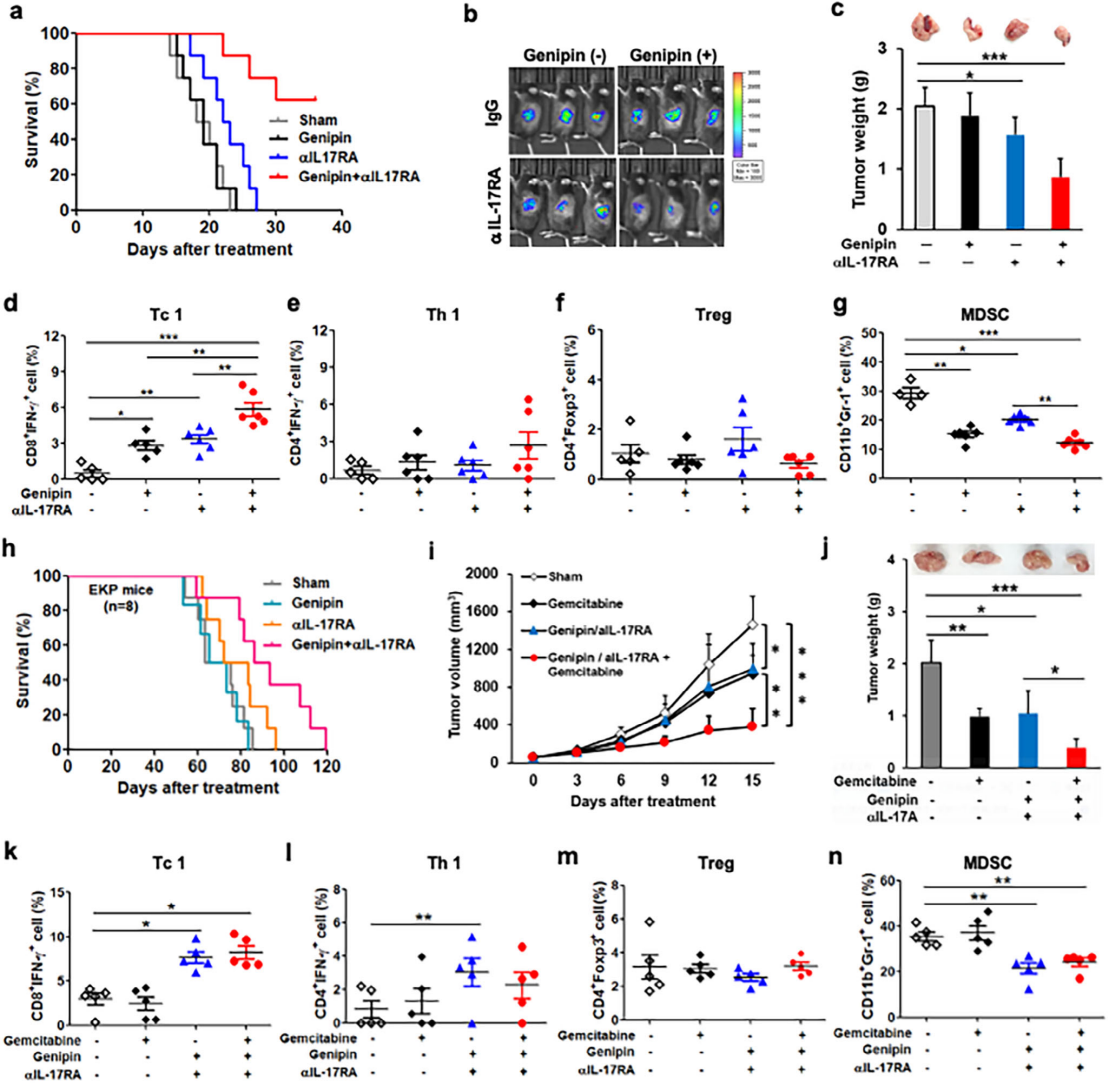
Dual UCP2 and IL‐17 blockade suppresses PDAC progression and enhances chemotherapy efficacy. Wildtype mice underwent orthotopic implantation of Pan18 cells and were treated with genipin (10 mg/kg, i.p., every two days) and αIL‐17RA (1 mg/kg, i.v., once weekly) for 3 weeks. (a–c) Overall survival (*n* = 8; a), in vivo bioluminescent tumor burden (*n* = 5–6; b), and tumor weight at endpoint (*n* = 5–6; c) are shown. (d–g) Flow‐cytometric quantification of intratumoral Tc1 (d), Th1 (e), Treg (f), and MDSC (g) populations (*n* = 5–6). Survival benefit was further validated in transgenic EKP mice treated with genipin and αIL‐17RA (*n* = 8; h). (i–n) Chemotherapy combination studies in orthotopic PDAC‐bearing mice. Genipin and αIL‐17RA were administered with or without gemcitabine (100 mg/kg, i.p., every 3 days) for 3 weeks (*n* = 5). Tumor volume (i) and tumor weight (j) were measured. (k–n) Flow‐cytometric analysis of intratumoral Tc1 (k), Th1 (l), Treg (m), and MDSC (n) populations (*n* = 5). Data are presented as mean ± SEM. ^*^ *p* < 0.05; ^**^ *p* < 0.01; ^***^ *p* < 0.001.

To further support translational relevance, we administered genipin together with anti‐IL‐17RA monoclonal antibody in PDAC models. Combined UCP2/IL‐17 targeting markedly suppressed tumor growth and improved survival (Figure 5a–c), with increased Tc1 infiltration and decreased MDSCs relative to monotherapies (Figure 5d–g). Similar effects were observed in a KPC‐2 subcutaneous model (Figure S7a–g) and an inducible Kras^G12D^ PDAC model (Figure 5h). Dual blockade also synergized with gemcitabine to enhance tumor control and boost immune effector signatures (Figure 5i–n).

**FIGURE 6:**
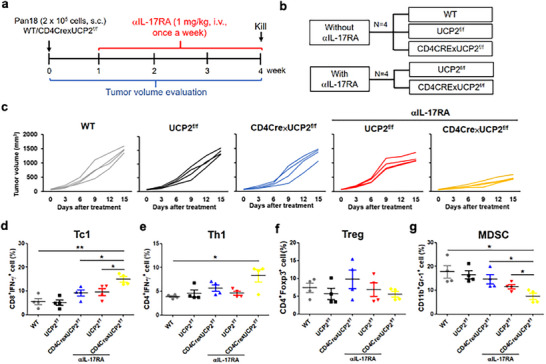
Conditional UCP2 deletion in CD4/CD8 T cells synergizes with IL‐17 blockade to suppress PDAC progression. Wild‐type and CD4Cre × UCP2^f/f^ mice were implanted subcutaneously with Pan18 cells and treated with αIL‐17RA (1 mg/kg, i.v., once weekly) for 3 weeks. Shown are schematic timeline and treatment groups (a,b), tumor volume kinetics (c), and flow‐cytometric quantification of intratumoral Tc1 (d), Th1 (e), Treg (f), and MDSC (g) populations (*n* = 4). Data represent mean ± SEM. ^*^ *p* < 0.05; ^**^ *p* < 0.01; ^***^ *p* < 0.001.

To genetically validate these findings, CD4Cre × UCP2^f/f^ mice were generated. Conditional T‐cell‐specific UCP2 deletion combined with anti‐IL‐17RA synergistically suppressed tumor growth, enhanced Tc1/Th1 accumulation, and reduced tumoral MDSCs (Figure 6a‐g). Dual blockade also augmented Tc1 responses in lymph nodes and spleen and increased circulating IFN‐γ and IL‐12 (Figure S8a,b). Notably, UCP2 deletion did not increase Th17 frequencies, supporting that IL‐17 elevation seen with pharmacologic UCP2 inhibition is TME‐dependent rather than T‐cell intrinsic. Finally, in a melanoma model characterized by high UCP2 expression, dual UCP2/IL‐17 blockade recapitulated the antitumor synergy observed in PDAC (Figure S9a–f), demonstrating tumor‐type‐agnostic efficacy.

### 2.6 CD8⁺ T Cell Depletion Abolishes the Therapeutic Benefit of Combined UCP2 and IL‐17 Blockade

**FIGURE 7:**
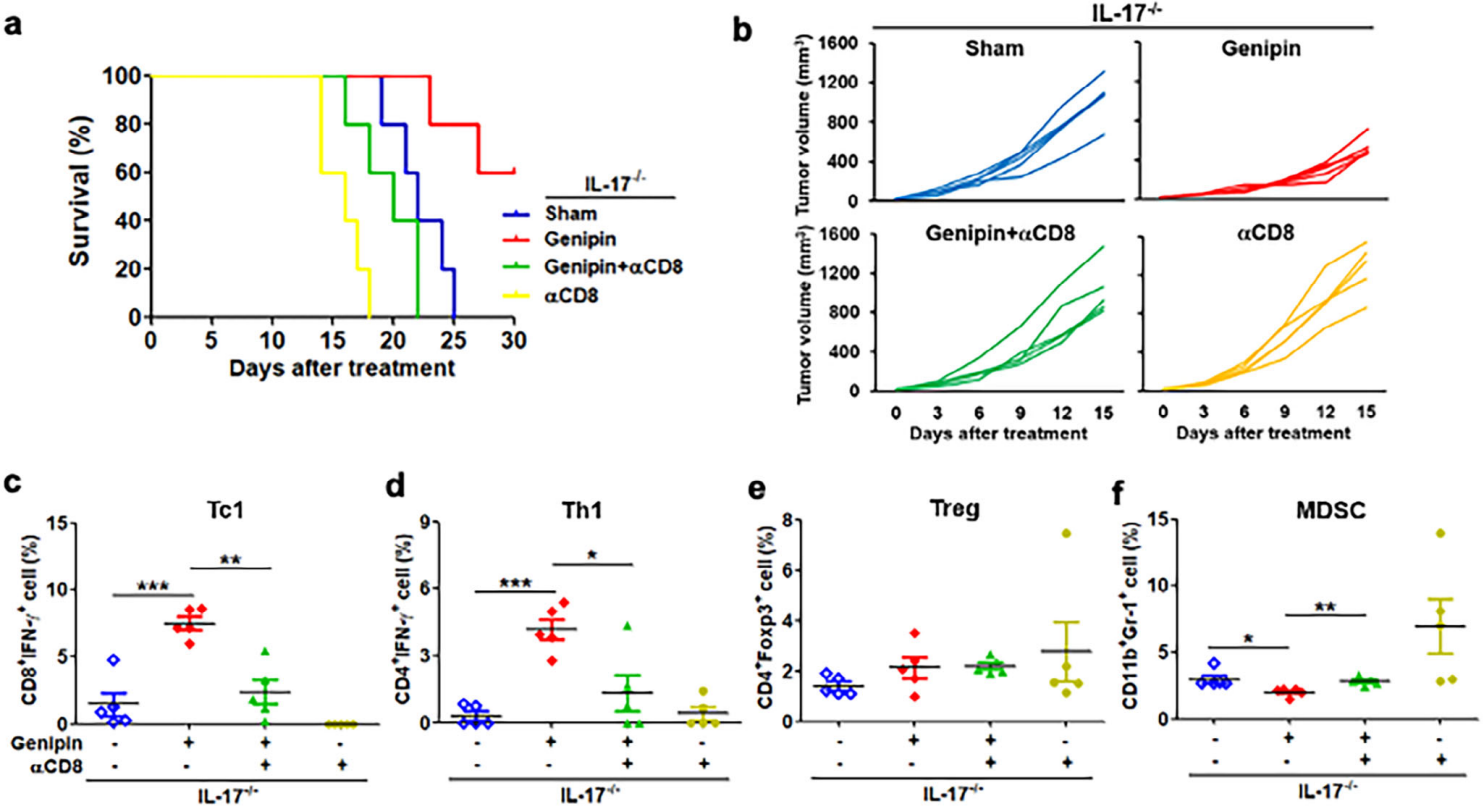
CD8⁺ T cell depletion abrogates the therapeutic benefit of UCP2 inhibition combined with IL‐17 blockade in PDAC. PDAC‐bearing IL‐17^−^/^−^ mice were subcutaneously implanted with Pan18 cells and treated with genipin (10 mg/kg, i.p., every two days) together with a CD8‐neutralizing antibody (5 mg/kg, i.v., weekly) for 3 weeks. Shown are overall survival (*n* = 5; a) and tumor volume (*n* = 5; b). Flow‐cytometric analyses of intratumoral Tc1 (c), Th1 (d), Treg (e), and MDSC (f) populations. Data are presented as mean ± SEM. ^*^ *p* < 0.05; ^**^ *p* < 0.01; ^***^ *p* < 0.001.

To further establish that the antitumor efficacy of UCP2 targeting is mediated predominantly through CD8⁺ T cells, we depleted CD8⁺ T cells in PDAC‐bearing IL‐17^−/−^ mice treated with genipin. CD8 neutralization completely abrogated the therapeutic benefit of UCP2 inhibition, resulting in accelerated tumor progression and shortened survival (Figure 7a,b). Loss of CD8⁺ T cells also reversed the immunologic remodeling induced by dual blockade, markedly reducing intratumoral Tc1 and Th1 infiltration while restoring MDSC accumulation (Figure 7c–f).

Together with our genetic UCP2‐knockout data, these results validate CD8⁺ T cells as indispensable mediators of UCP2‐driven antitumor immunity and demonstrate that UCP2 blockade exerts its therapeutic effect by potentiating cytotoxic effector responses rather than broadly modulating immune subsets.

### 2.7 UCP2 suppression Enhances IFN‐γ Production in Peripheral and Tumor‐Infiltrating T Cells From Patients With PDAC

**FIGURE 8:**
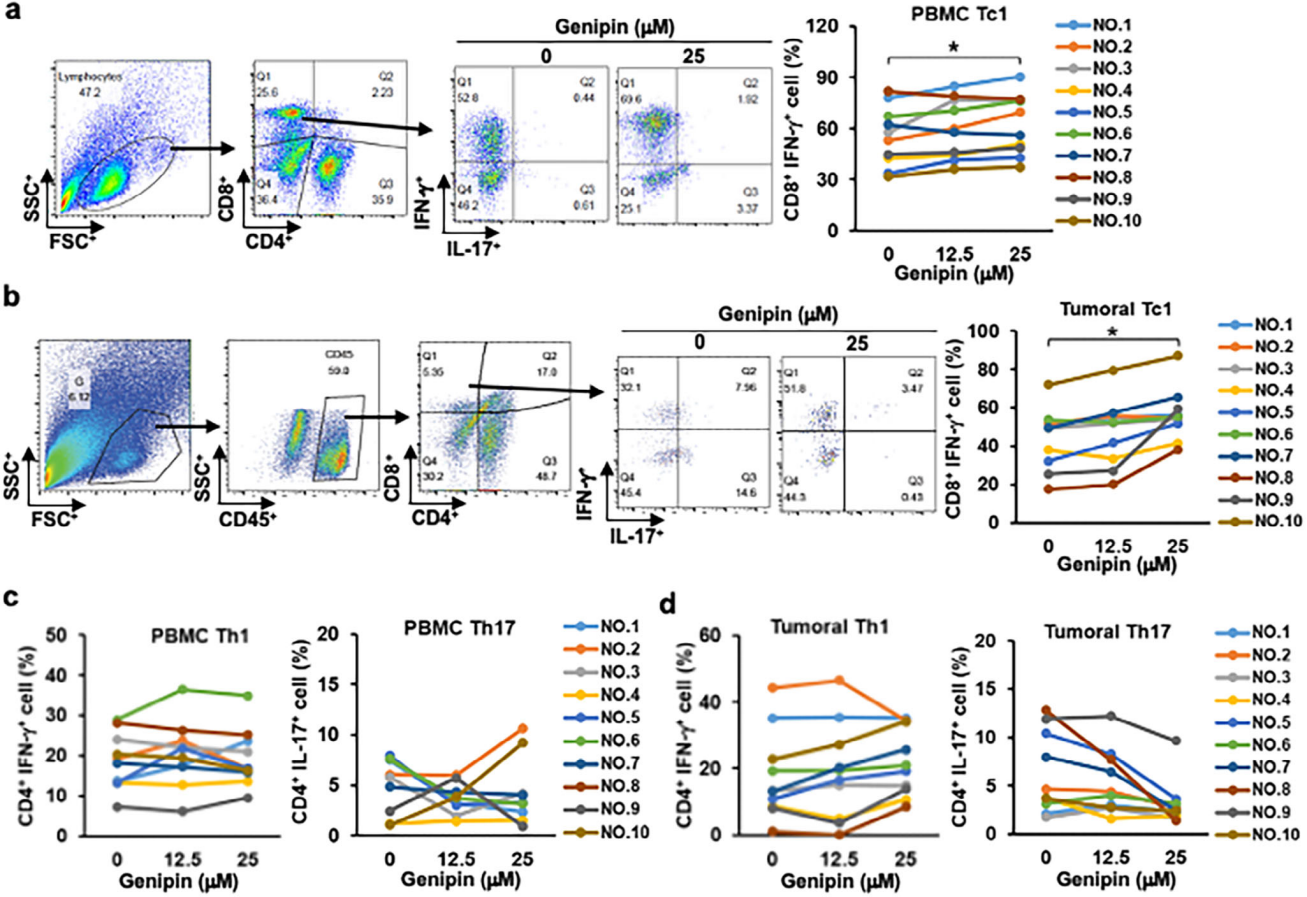
UCP2 inhibition enhances IFN‐γ production in circulating and tumor‐infiltrating T cells from patients with PDAC. Peripheral blood mononuclear cells (PBMCs) and dissociated tumor specimens from patients with PDAC (*n* = 10) were cultured ex vivo and treated overnight with genipin before flow‐cytometric analysis. (a,b) Gating strategy, representative dot plots, and cumulative quantification of IFN‐γ–producing CD8⁺ T cells in genipin‐treated PBMCs (a) and tumor‐derived T cells (b). (c,d) Cumulative quantification of IFN‐γ–producing and IL‐17–producing CD4⁺ T cells in genipin‐treated PBMCs (c) and tumor samples (d). Data are presented as mean ± SEM. ^*^ *p* < 0.05; ^**^ *p* < 0.01; ^***^ *p* < 0.001.

To evaluate the translational relevance of UCP2 targeting in human disease, we examined the effect of UCP2 inhibition in peripheral blood and tumor‐infiltrating T cells from patients with PDAC. Consistent with our murine studies, pharmacologic UCP2 blockade significantly increased IFN‐γ–producing CD8⁺ T cells in both peripheral blood mononuclear cells (PBMCs) and matched tumor specimens (Figure 8a,b). In contrast, Th1 frequencies remained unchanged in either compartment (Figure 8c,d), suggesting a preferential enhancement of cytotoxic effector responses. Notably, UCP2 inhibition did not induce Th17 expansion in PBMCs or tumors from patients with PDAC (Figure 8c,d), aligning with our genetic models and supporting that the IL‐17 upregulation observed during genipin treatment in vivo is context‐dependent and driven by the tumor microenvironment rather than by intrinsic T‐cell programming.

Together, these findings validate the human applicability of UCP2 targeting and provide clinical evidence that UCP2 inhibition selectively augments cytotoxic T‐cell function without promoting pathogenic Th17 responses in patients with PDAC.

## 3 Discussion

Pancreatic ductal adenocarcinoma remains a highly lethal malignancy, with most patients experiencing rapid progression and a median survival of only 4–6 months after diagnosis. Although frontline chemotherapy offers modest survival benefit, its toxicity limits broad use, particularly in patients with advanced disease burden [17]. Immunotherapy has revolutionized treatment for several solid tumors; however, its efficacy in PDAC has been severely limited. This reflects the complex interplay between tumor cells and the desmoplastic, immunosuppressive TME, which restricts effector lymphocyte infiltration and function.

In this study, we define the therapeutic potential of a combinatorial immunometabolic approach in PDAC. We demonstrate that UCP2 targeting enhances type I effector responses by upregulating the IL‐12R/STAT4/mTOR axis and promoting T‐bet–driven IFN‐γ production in Tc1 cells. In vivo, genetic or pharmacologic UCP2 inhibition enhanced antitumor immunity and attenuated PDAC‐induced immunosuppression. Importantly, IL‐17 blockade synergized with UCP2 inhibition, re‐shaping chemokine signaling to favor Tc1 infiltration while constraining MDSC accumulation. Together, these effects resulted in robust tumor control across multiple preclinical PDAC models, including orthotopic and xenograft systems, supporting this strategy as a rational immunotherapeutic avenue for a classically immunoresistant malignancy.

Previous studies have implicated UCP2 in CD8⁺ T‐cell metabolism during tumor immunity [18]. Here, we extend this knowledge by establishing UCP2 as a key regulator of Tc1 metabolic fitness within the hostile PDAC TME. Mechanistically, UCP2 blockade enhanced mitochondrial oxidative metabolism, increased acetyl‐CoA availability, and promoted histone acetylation, all of which support sustained IFN‐γ expression in activated effector T cells [12, 19]. We further identify a selective role for UCP2 in limiting mitochondrial pyruvate flux via MPC, without affecting total pyruvate production, thereby linking UCP2 activity to metabolic checkpoint control during Tc1 activation.

While immune‐intrinsic UCP2 suppressed antitumor immunity, emerging evidence suggests context‐dependent roles for tumor‐intrinsic UCP2 in modulating TME and promoting antitumor immune responses [20]. We speculate that these divergent outcomes reflect tumor‐type‐specific metabolic architectures, consistent with prior observations of context‐dependent UCP2 signaling in cancer biology [21, 22, 23]. Our findings support a model in which UCP2‐driven metabolic reprogramming is essential for Tc1 persistence and cytotoxicity, while simultaneously sensitizing PDAC cells to oxidative cell death, thereby producing dual immunologic and tumor‐cell‐intrinsic benefits.

Our study further establishes the detrimental impact of IL‐17 on anti‐tumor immunity in PDAC, primarily through impaired Tc1 recruitment and maintenance of immunosuppressive MDSC populations. We identify IL‐17‐driven chemokine dysregulation as a central mechanism, characterized by reduced CXCL10 and increased CCL2 expression—both recognized IL‐17‐regulated targets in prior reports [24, 25, 26]. Notably, while UCP2 inhibition did not elevate IL‐17 in purified Th17/Tc17 subsets in vitro, in vivo treatment led to a marked increase in IL‐17A⁺ T cells within the TME. This likely reflects extrinsic cues from myeloid and stromal populations secreting IL‐1β, IL‐6, and TGF‐β under conditions of metabolic stress and ROS elevation [27]. Such context‐dependent IL‐17 induction reinforces that genipin effects are not T‐cell intrinsic but instead shaped by inflammatory and metabolic cues unique to PDAC.

Recent work demonstrates that tumors co‐opt metabolic and cytokine networks to subvert CD8⁺ cytotoxicity; for example, ribonuclease‐1–mediated STAT1 hijacking was shown to drive T‐cell dysfunction and suppress IFN‐γ and CXCL10 expression [28]. This aligns with our findings that IL‐17‐rich environments suppress type I immunity and confirms the rationale for concomitant UCP2 and IL‐17 blockade. Further, IL‐17 acts through paracrine mechanisms to expand and recruit MDSCs, sustaining immunosuppressive niches that facilitate tumor progression, metastasis, and Tc1 dysfunction [29, 30]. This paradigm is particularly relevant in PDAC, where a dense desmoplastic stroma amplifies immunologic exclusion and may explain the poor efficacy of single‐agent immunotherapies.

Although IL‐17 blockade alone produced limited effects, its combination with UCP2 inhibition enhanced Tc1 activation ex vivo using PDAC patient samples and synergized with standard chemotherapy in vivo, indicating broad translational potential. These findings align with efforts to reprogram the pancreatic TME to alleviate immune suppression [3]. Collectively, our data highlight that simultaneous targeting of cytokine‐driven immune suppression and metabolic control mechanisms can overcome key resistance barriers in PDAC. Given that IL‐17 inhibitors are already clinically approved for autoimmune disorders, our work provides a compelling rationale for clinical investigation of dual UCP2/IL‐17 targeting strategies to augment immunotherapy responses in this otherwise treatment‐refractory malignancy.

## 4 Experimental Section

### 4.1 Mice

IL‐17 knockout mice (generously provided by Dr. Yoichiro Iwakura, University of Tokyo, Japan) and C57BL/6 mice (purchased from the National Laboratory Animal Center, Taiwan) were used at 7–10 weeks of age. Animals were housed under specific pathogen‐free (SPF) conditions in the Animal Center of China Medical University, Taiwan. All procedures were performed in accordance with institutional guidelines and approved by the Institutional Animal Care and Use Committee of China Medical University, Taichung, Taiwan (CMUIACUC‐2022‐080).

For conditional UCP2 deletion in T cells, CD4Cre × UCP2^f/f^ mice were generated by crossing CD4Cre mice with loxP‐UCP2‐loxP mice (constructed using CRISPR/Cas9 technology; Taiwan Mouse Clinic, National Science and Technology Council, Taiwan). Mice were randomly assigned to treatment groups, and investigators were blinded to treatment allocation during tumor measurements and analysis.

### 4.2 Cells

The human pancreatic cancer cell lines MIA PaCa‐2 (RRID: CVCL_0428), PANC‐1 (RRID: CVCL_0480), and SU.86.86 (RRID: CVCL_3893) were generously provided by Prof. Wen‐Hwa Lee (Academia Sinica, Taiwan) and maintained in complete growth medium in a humidified incubator at 37°C with 5% CO_2_. The murine PDAC cell line Pan18 (GFP‐LUC‐tagged), derived from pancreatic tumors in EKP (Elastase‐CreER; LSL‐Kras^G12D^; p53^+/−^) C57BL/6 mice, was kindly provided by Dr. Chia‐Ning Shen (Academia Sinica, Taiwan) and used for syngeneic tumor implantation [31]. The KPC‐2 cell line (GFP‐LUC‐tagged), derived from pancreatic tumors in PDX‐Cre; LSL‐Kras^G12D^; p53^f/f^ C57BL/6 mice, was established by Prof. Heng‐Hsiung Wu and used for syngeneic mouse modeling. Pan18 and KPC‐2 cell lines, generated in‐house or through academic collaboration, do not have RRIDs assigned in public repositories; however, their genetic background, tumorigenic features, and reporter expression profiles have been characterized and further validated in our study. All cell lines tested negative for mycoplasma contamination prior to use, confirmed using the MorreMyco Mycoplasma Detector kit (#MMMD50, Quantum Biotechnology Inc., Taiwan).

### 4.3 Genipin Preparation

Genipin stock solution (100 mM in DMSO) was freshly diluted into complete medium to final working concentrations of 6.25, 12.5, or 25 µM. Corresponding DMSO controls (0.00625%, 0.0125%, and 0.025% v/v) were included to match vehicle exposure. These DMSO concentrations are below cytotoxicity thresholds reported for primary lymphocytes and tumor‐cell cultures and did not affect cell viability under our experimental conditions.

### 4.4 In Vitro CD4 or CD8 T Cell Culture

Mouse lymph nodes and spleens were harvested, and naïve CD4 or CD8 T cells were isolated by magnetic bead separation (STEMCELL Technologies). Purified T cells were plated in 96‐well plates pre‐coated with anti‐CD3 (10 µg/mL) and anti‐CD28 (1 µg/mL) and stimulated under the following skewing conditions: Th/Tc1 cells were polarized with IL‐12 (3 ng/mL) and anti‐IL‐4 (10 µg/mL), Th/Tc2 cells with IL‐4 (20 ng/mL) and anti‐IFN‐γ (10 µg/mL), Th/Tc17 cells with IL‐6 (20 ng/mL), TGF‐β (1.25 ng/mL), IL‐1β (20 ng/mL), IL‐23 (20 ng/mL), anti‐IFN‐γ (10 µg/mL), and anti‐IL‐4 (10 µg/mL), and Treg cells with TGF‐β (5 ng/mL), anti‐IFN‐γ (10 µg/mL), and anti‐IL‐4 (10 µg/mL). Cells were cultured for 4–5 days and subsequently treated with genipin (3.125, 6.25, or 12.5 µM) or an equivalent volume of DMSO for 12–24 h prior to downstream analysis. For genetic validation of UCP2 function, Tc1 cells were cultured under the same polarization conditions as described above, except that CD8⁺ T cells were isolated from WT or CD4Cre × UCP2^f/f^ mice prior to activation.

### 4.5 Surface Marker and Intracellular Cytokine Staining

Monoclonal antibodies were obtained from BD Biosciences or eBioscience. For intracellular cytokine staining (ICS), cells were restimulated by PMA and ionomycin for 5 h in the presence of GolgiStop (BD Biosciences), followed by surface staining, subsequent fixed and permeabilized using the BD Cytofix/Cytoperm kit. Cells were then stained with antibodies against intracellular cytokines, fixed in 1.5% formaldehyde for 10 min at room temperature, and analyzed on a FACSVerse flow cytometer (BD Biosciences, San Diego, CA, USA). Data were processed using FlowJo software (Tree Star Inc.).

### 4.6 Gene Expression Analysis

Expression profiles of UCP2 and single‐cell transcriptomic distributions across human tissues, normalized to transcripts per million protein‐coding genes (pTPM), were obtained from the Human Protein Atlas (HPA) database. For RNA sequencing, total RNA from genipin‐treated and control Tc1 cells was processed and analyzed by Genomics (Taiwan) following Illumina workflows. Raw reads were subjected to adapter trimming and low‐quality base removal, and library quality was assessed using the Agilent Bioanalyzer 2100 system. Differential gene expression was determined using EBSeq, and functional enrichment (GO analysis) was performed with clusterProfiler. For quantitative real‐time PCR, total RNA was isolated using the RNeasy Micro Kit (Qiagen), and cDNA was synthesized using the PrimeScript RT reagent kit (TaKaRa). IFN‐γ transcript levels were quantified using TaqMan Gene Expression Assay (Mm01168134_m1) on a StepOnePlus Real‐Time PCR System (Applied Biosystems), with 18S serving as the endogenous control. Relative expression levels were calculated using the 2^−ΔΔCt^ method.

### 4.7 Immunoblotting

Protein concentrations of the whole‐cell lysate or purified histones were quantified using the Bradford assay (Bio‐Rad Laboratories). Equal amounts of protein were separated by 10% SDS–PAGE and transferred to PVDF membranes, followed by overnight incubation with primary antibodies (1:1000 dilution) and subsequent incubation with HRP‐conjugated secondary antibodies (1:5000; GeneTex). Membranes were probed with antibodies against UCP2, p‐PI3K/PI3K, p‐AKT/AKT, p‐mTOR/mTOR, p‐STAT1/STAT1, p‐STAT4/STAT4, and H3K9ac (Cell Signaling Technology or GeneTex). Actin or Lamin B1 (Merck‐Millipore, Darmstadt, Germany) served as loading controls. Protein signals were detected using enhanced chemiluminescence (GE Healthcare), and band intensities were quantified using ImageJ software.

### 4.8 Metabolic Assays

Real‐time extracellular acidification rate (ECAR) and oxygen consumption rate (OCR) were measured using a Seahorse XF24 analyzer (Seahorse Bioscience) with Glycolysis Stress Test and Mito Stress Test kits to assess glycolytic and oxidative metabolism. Briefly, 5 × 10^5^ cells per well were plated in Cell‐Tak–coated XF‐24 microplates (Corning) 24 h prior to analysis. ECAR and OCR were recorded following sequential injections of oligomycin, FCCP, 2‐deoxy‐D‐glucose (2‐DG), and rotenone/antimycin A. Data were analyzed using Agilent Seahorse Wave software.

For mitochondrial isolation, Tc1 cells were freeze‐thawed to weaken membranes and resuspended in Reagent A from a mitochondria isolation kit (ab110170, Abcam), followed by homogenization and centrifugation at 1,000 × g for 10 min at 4°C. The supernatant (SN#1) was collected, and the pellet was resuspended in Reagent B, homogenized, and centrifuged again to obtain SN#2. SN#1 and SN#2 were combined and centrifuged at 12,000 × g for 15 min at 4°C to isolate mitochondria. The mitochondrial pellet was resuspended in Reagent C containing protease inhibitors and used for metabolite quantification. Mitochondrial pyruvate and acetyl‐CoA levels were measured using the Pyruvate Assay Kit (ab65342, Abcam) and PicoProbe Acetyl‐CoA Assay Kit (ab87546, Abcam) according to the manufacturer's protocols.

### 4.9 Histone H3 and H4 Total Acetylation

Histones were extracted from Tc1 cells using the EpiSeeker Histone Extraction Kit (ab113476; Abcam, Cambridge, UK) according to the manufacturer's instructions. Total acetylation levels of histone H3 and histone H4 were quantified using the H3/H4 Acetylation Detection Fast Fluorometric Kits (ab131561 and ab131562, Abcam), following the manufacturer's protocols.

### 4.10 Soft Agar Colony Formation

Anchorage‐independent growth was assessed by seeding 2000–4000 cells per well in 12‐well plates in a top layer of 0.35% agar in complete medium over a 0.5% agar base. Cultures were maintained with 50 µL serum‐free medium containing genipin replenished every 3 days. After 14–21 days, colonies were fixed and stained with crystal violet (Sigma–Aldrich, C3886) and counted under a light microscope.

### 4.11 Invasion Assay

Cell invasion was evaluated using Matrigel‐coated Transwell inserts (Millicell, PET 8 µm; Millipore). Cells were seeded in the upper chamber in serum‐free media ± genipin, and complete medium was added to the lower chamber as chemoattractant. After 48–72 h, migrated cells were fixed in formaldehyde, stained with crystal violet, and quantified microscopically.

### 4.12 Oxidative Stress and Apoptosis Assays

Intracellular ROS, lipid peroxidation, nitric oxide (NO), and glutathione peroxidase (GPx) activity in PDAC cells were measured using the respective assay kits from Biovision (Milpitas, CA, USA) according to the manufacturer's instructions. Apoptosis was quantified using a FITC‐Annexin V/PI kit (BD PharMingen, San Diego, CA, USA) and analyzed on a FACSVerse flow cytometer (BD Biosciences, San Diego, CA, USA) [32, 33].

### 4.13 Tumorigenicity Assay in Mice

For subcutaneous Pan18 implantation, 2 × 10^5^ Pan18 cells were injected into wild‐type (WT) or IL‐17^−/−^ mice. Genipin (5, 10, or 30 mg/kg, intraperitoneally [i.p.], every 2 days) was administered for 3 weeks. For subcutaneous KPC‐2 implantation, 2 × 10^5^ KPC‐2 cells were injected into WT mice, followed by genipin (10 mg/kg, i.p., every 2 days) and recombinant α‐IL‐17RA (1 mg/kg, intravenous [i.v.], once weekly) for 3 weeks. Tumor volumes were measured every 2 days.

For orthotopic PDAC modeling, 2 × 10^5^ Pan18 cells were injected into the pancreas. Tumor growth was monitored weekly using an IVIS imaging system (Caliper Life Sciences). Genipin (10 mg/kg, i.p., every 2 days) and α‐IL‐17RA (1 mg/kg, i.v., weekly) were administered starting 1‐week post‐implantation. Mice were sacrificed at day 35 for tumor collection and weighing.

For the spontaneous PDAC model, Kras+/LSL‐G12D mice (B6;129‐Kras2) [34] were crossed with Elas‐CreER mice [35, 36] to generate Elas‐CreERT; Kras+/LSL‐G12D mice.

To induce oncogenic Kras expression in acinar cells, 5‐week‐old male mice received tamoxifen (20 mg/mL, 100 µL, i.p., three times weekly) for 1 week, followed by cerulein (50 µg/mL, 100 µL, i.p., six times weekly; BACHEM) for 3 weeks. Genipin (10 mg/kg, i.p., every 2 days) and α‐IL‐17RA (1 mg/kg, i.v., weekly) were administered thereafter, and survival was monitored.

For chemo‐combination studies, PDAC‐bearing mice received genipin (10 mg/kg, i.p., every 2 days) and α‐IL‐17RA (1 mg/kg, i.v., weekly), with or without gemcitabine (100 mg/kg, i.p., every 3 days; Cayman Chemical) for 3 weeks.

For T‐cell–specific UCP2 deletion studies, CD4Cre × UCP2^f/f^ mice were generated by crossing CD4Cre mice with loxP‐UCP2‐loxP mice. CD4Cre × UCP2^f/f^, WT, and loxP controls received subcutaneous 2 × 10^5^ Pan18 implantation followed by α‐IL‐17RA (1 mg/kg, i.v., weekly) for 3 weeks.

For the melanoma model, IL‐17^−/−^ and WT mice were injected subcutaneously with 5 × 10^5^ B16F10 cells, followed by genipin (10 mg/kg, i.p., every 2 days) for 3 weeks.

For CD8‐depletion studies, IL‐17^−/−^ mice were implanted subcutaneously with 2 × 10^5^ Pan18 cells and treated with genipin (10 mg/kg, i.p., every 2 days) plus CD8‐neutralizing antibody (5 mg/kg, i.v., weekly) for 3 weeks. Tumor volumes were monitored every 3 days.

For immunophenotyping, tumors were minced and digested in RPMI‐1640 containing collagenase IV, DNase I, and hyaluronidase for 30 min at 37°C. Cell suspensions were filtered through 70‐µm strainers and analyzed by flow cytometry for Tc1, Th1, Treg, and MDSC subsets.

### 4.14 Serum Cytokines Analysis

Serum IFN‐γ, IL‐17, TNF‐α, IL‐12, IL‐6, and TGF‐β concentrations were quantified using commercial ELISA kits (BD Biosciences; R&D systems; BioLegend) following manufacturer instructions.

### 4.15 Chemokine Profiling

Tumor lysates were quantified by Bradford assay, and 250 µg protein per sample was analyzed using a Proteome Profiler Mouse Chemokine Array Kit (ARY020; R&D Systems) per manufacturer protocols.

### 4.16 Immunohistochemistry

Formalin‐fixed paraffin‐embedded tumor tissues were sectioned and subjected to heat‐induced antigen retrieval in citrate buffer (pH 6.0) using autoclave treatment for 15 min. Sections were blocked in PBS containing 5% FBS and incubated overnight at 4°C with primary antibodies against CXCL10 (1:200, GTX31179, GeneTex), CCL2 (1:200, GTX81767, GeneTex), 4‐hydroxynonenal (4‐HNE; 1:200, ab46545, Abcam), and CD3 (1:250, ab16669, Abcam). Signal detection was performed using the UltraVision HRP/DAB detection system (Thermo Fisher Scientific). Nuclei were counterstained with hematoxylin, dehydrated, and mounted. Stained slides were imaged using a Nikon Eclipse E600 microscope, and six random high‐power fields per sample were captured for quantitative analysis (Image‐Pro Plus 4.0).

### 4.17 Human Subjects and Ex Vivo T‐Cell Assays

Human PDAC specimen collection was approved by the Research Ethics Committee of China Medical University & Hospital, Taichung, Taiwan (CMUH109‐REC2‐148), and informed consent was obtained from all participants (Table S2). Fresh peripheral blood (10 mL) and matched tumor tissue (0.1–0.5 mm^3^) were collected at the time of surgical resection. PBMCs and tumor‐infiltrating immune cells were isolated by density‐gradient centrifugation or mechanical dissociation, respectively. Cells were cultured in RPMI‐1640 supplemented with 10% FBS at 2 × 10^5^ cells/well in 12‐well plates and treated with genipin (12.5 or 25 µM) overnight prior to flow‐cytometric analysis.

### 4.18 Statistical Analysis

Data are presented as mean ± SEM. Normality was assessed by the Shapiro–Wilk test. For two‐group comparisons, a two‐tailed unpaired Student's *t*‐test was used. For comparisons involving more than two groups, one‐way ANOVA followed by Tukey's post hoc test was applied. *, **, and *** indicate statistical significance at *p* < 0.05, *p* < 0.01, and *p* < 0.001, respectively. Sample sizes (n) are provided in figure legends. Analyses were performed using GraphPad Prism v9.0 (GraphPad Software).

## Author Contributions

H.R.Y. conceived and supervised the project. C.T.L., Y.C.S., and H.R.Y. designed the project. C.T.L., H.H.W., T.C.W., C.H.L., Y.C.S., and H.R.Y. performed experiments. Y.I., Y.J.D., and C.D. provided the IL‐17 knockout mice. V.R. conceived the project. C.C.Y., T.C.W., Y.T.K., and C.T.L. collected the human samples and clinical data. C.T.L. analyzed all of the data. C.H.L., V.R., Y.C.S., and H.R.Y. prepared the manuscript. Y.C.S., H.H.W., and H.R.Y. contributed equally as co‐corresponding authors. All authors read the manuscript, provided feedback, and approved the final manuscript.

## Conflicts of Interest

The authors declare no conflict of interest.

## Supporting information




**Supporting File**: advs73450‐sup‐0001‐SuppMat.docx.

## Data Availability

The data that support the ﬁndings of this study are available from the corresponding author upon reasonable request.
