# Supplementary material for: Metabolic Reprogramming of T Cells by Dual UCP2 and IL‐17 Blockade Enhances Immunity Against Pancreatic Cancer
Source: Adv Sci (Weinh). 2026 Jan 4;13(16):e13020. doi: 10.1002/advs.202513020 (PMC13042516; doi:10.1002/advs.202513020)
Supplement: Supplementary file 1 — Supporting File: advs73450‐sup‐0001‐SuppMat.docx. [file ADVS-13-e13020-s001.docx]

**Supplementary Materials**

**
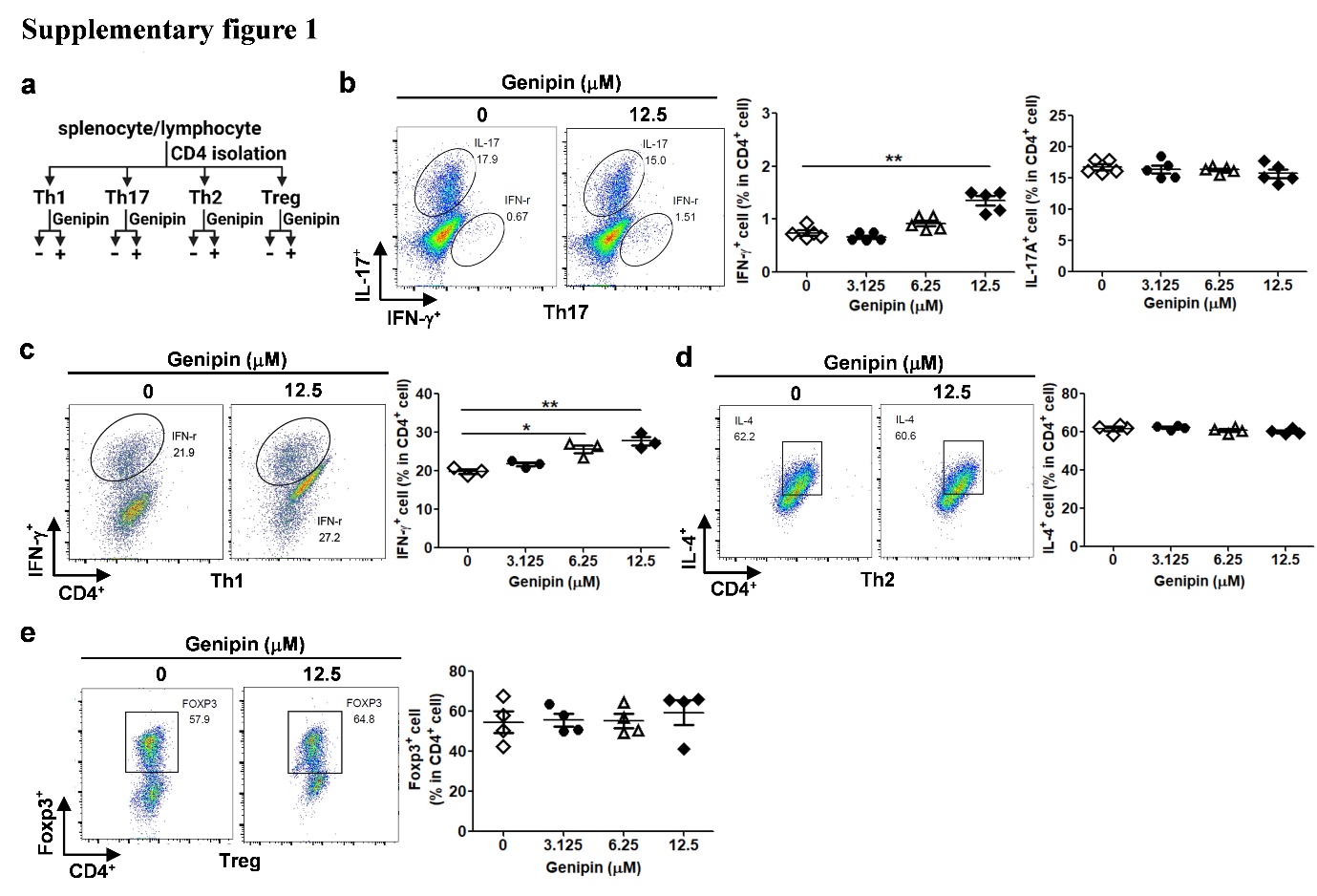
**

**Supplementary Figure S1. UCP2 inhibition enhances type-1 helper T-cell polarization without altering Th2 or Treg differentiation.** (a) Schematic of *in vitro* skewing and UCP2 blockade in CD4⁺ T-cell subsets. Polarized Th cells were treated overnight with genipin (3.125, 6.25, or 12.5 μM) and analyzed by intracellular cytokine staining (ICS). (b) Representative flow-cytometry plots and cumulative frequencies of IL-17⁺ and IFN-γ⁺ CD4⁺ T cells in genipin-treated Th17 cultures (N = 5). (c) Flow-cytometry plots and cumulative frequencies of IFN-γ⁺ CD4⁺ T cells in genipin-treated Th1 cultures (N = 3). (d) Flow-cytometry plots and cumulative frequencies of IL-4⁺ CD4⁺ T cells in genipin-treated Th2 cultures (N = 4). (e) Flow-cytometry plots and cumulative frequencies of Foxp3⁺ CD4⁺ T cells in genipin-treated Treg cultures (N = 4). Dot plots are gated on CD4⁺ lymphocytes. Data are presented as mean ± SEM. *P < 0.05; **P < 0.01.

**
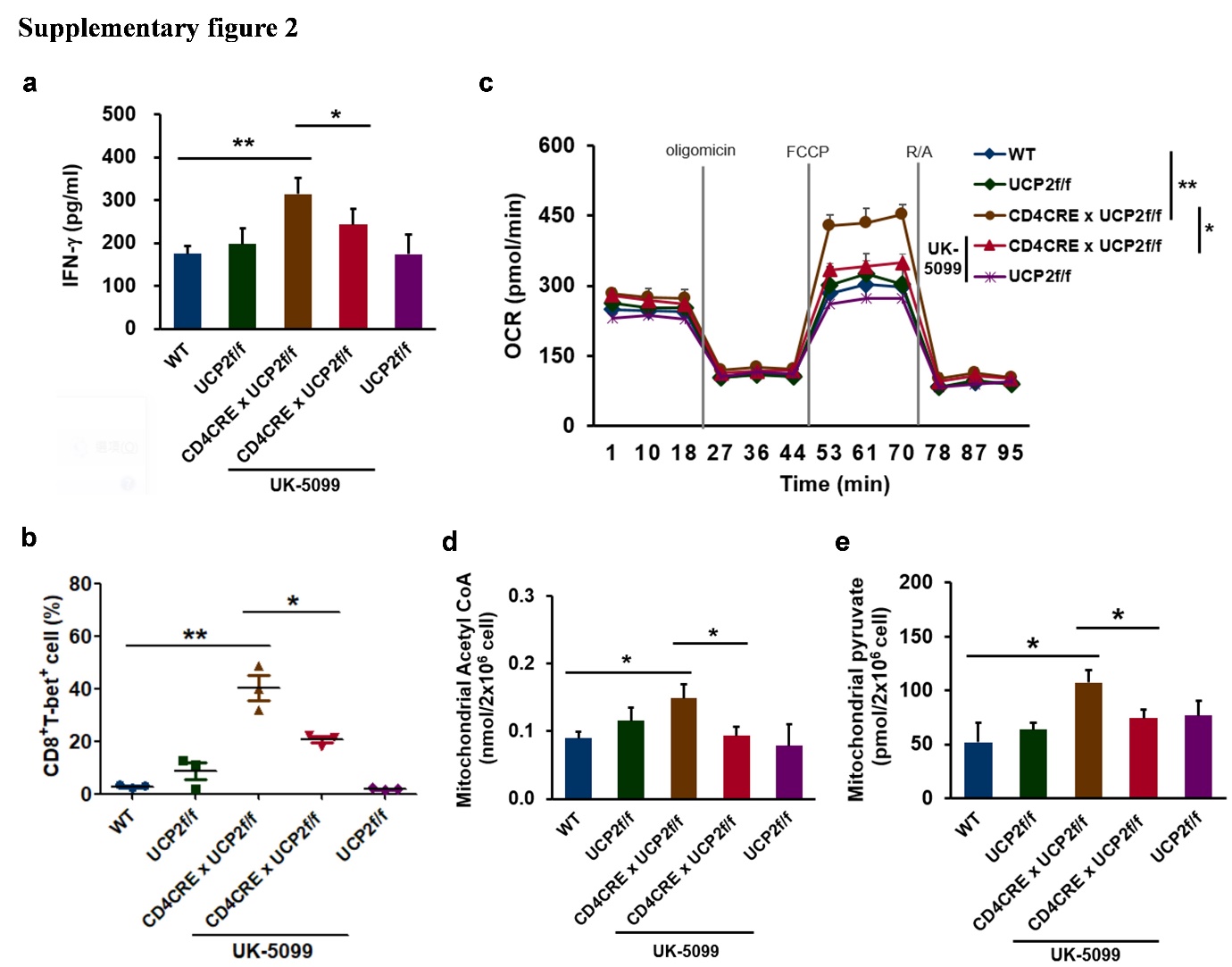
**

**Supplementary Figure S2. Genetic deletion of UCP2 enhances Tc1 effector function and mitochondrial metabolism in CD8⁺ T cells.** Naïve CD8⁺ T cells were isolated from WT or CD4Cre × UCP2^f/f^ mice, polarized under Tc1 conditions, and subsequently treated with or without UK-5099 (40 µM) (N = 3). (a) IFN-γ production and (b) T-bet expression were assessed by intracellular cytokine staining and ELISA. (c) Oxygen consumption rate (OCR) was quantified using a Seahorse XF analyzer. (d) Mitochondrial acetyl-CoA and (e) mitochondrial pyruvate levels in Tc1-polarized CD8⁺ T cells were measured by ELISA. Data are presented as mean ± SEM. *P < 0.05; **P < 0.01.

**
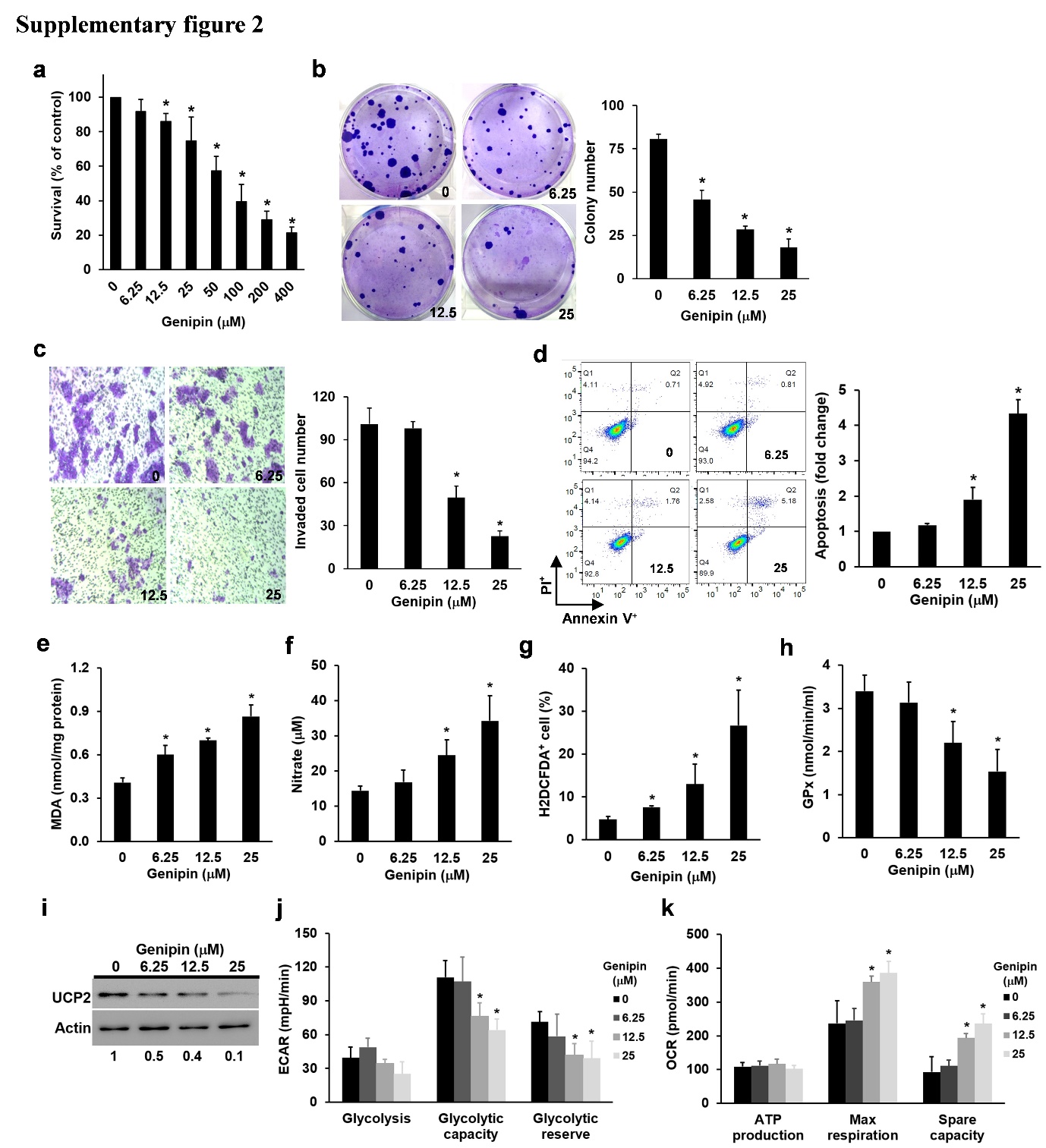
**

**Supplementary Figure S3. UCP2 inhibition suppresses tumorigenic and metastatic capacity of murine PDAC cells and induces oxidative stress.** (a) Viability of Pan18 cells treated with genipin for 24 h, assessed by MTT assay (N = 4). (b, c) Long-term clonogenicity and invasive capacity of Pan18 cells treated with genipin. Colony formation was quantified after 14 days of treatment (N = 4; b), and Matrigel invasion was assessed after 48 h (N = 4; c). (d) Apoptosis of Pan18 cells following 12 h genipin treatment. Representative flow-cytometry plots and frequencies of Annexin V⁺ cells relative to untreated controls (N = 4). (e–h) Oxidative stress markers in genipin-treated Pan18 cells. Intracellular lipid peroxidation (MDA, N = 4; e), nitrate (N = 4; f), ROS (H₂DCFDA, N = 4; g), and GPx activity (N = 4; h) were quantified. (i) UCP2 protein expression in Pan18 cells following genipin treatment; representative immunoblot and densitometric quantification. (j, k) Bioenergetic profiling of genipin-treated Pan18 cells. ECAR (N = 5; j) and OCR (N = 5; k) were measured by Seahorse XF24 analysis following metabolic inhibitor challenge. Data are presented as mean ± SEM. *P < 0.05 vs control.

**
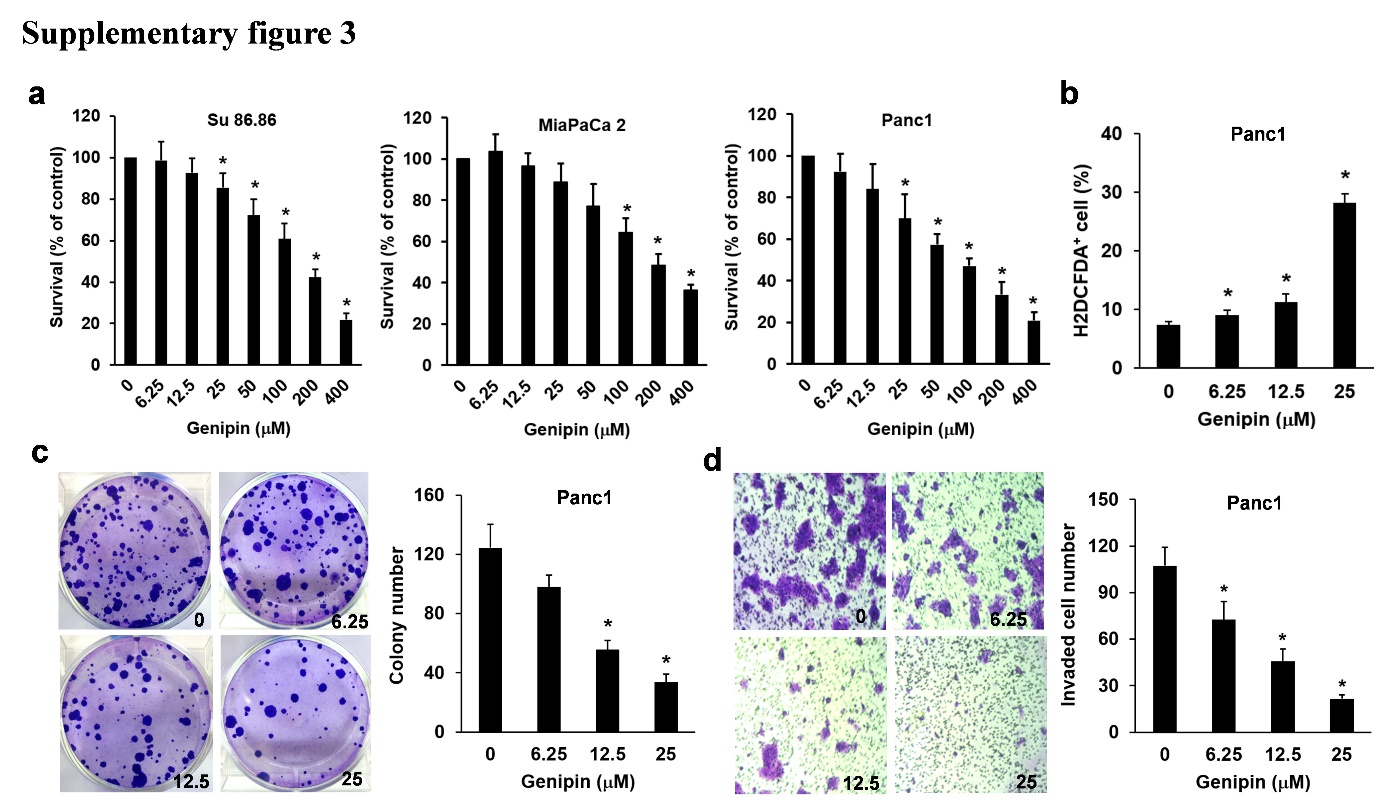
**

**Supplementary Figure S4. Antitumor and antimetastatic effects of UCP2 inhibition in human PDAC cells.** (a) Viability of genipin-treated human PDAC cell lines (Su.86.86, MiaPaCa-2, and PANC-1) after 24 h assessed by MTT assay (N=4). (b) Intracellular ROS levels (H2DCFDA) in genipin-treated PANC-1 cells. (c, d) Colony-forming ability (21-day assay; N=4) and invasion capacity (72-h assay; N=4) of PANC-1 cells following genipin exposure. Data are presented as mean ± SEM. *P < 0.05 versus control.

**
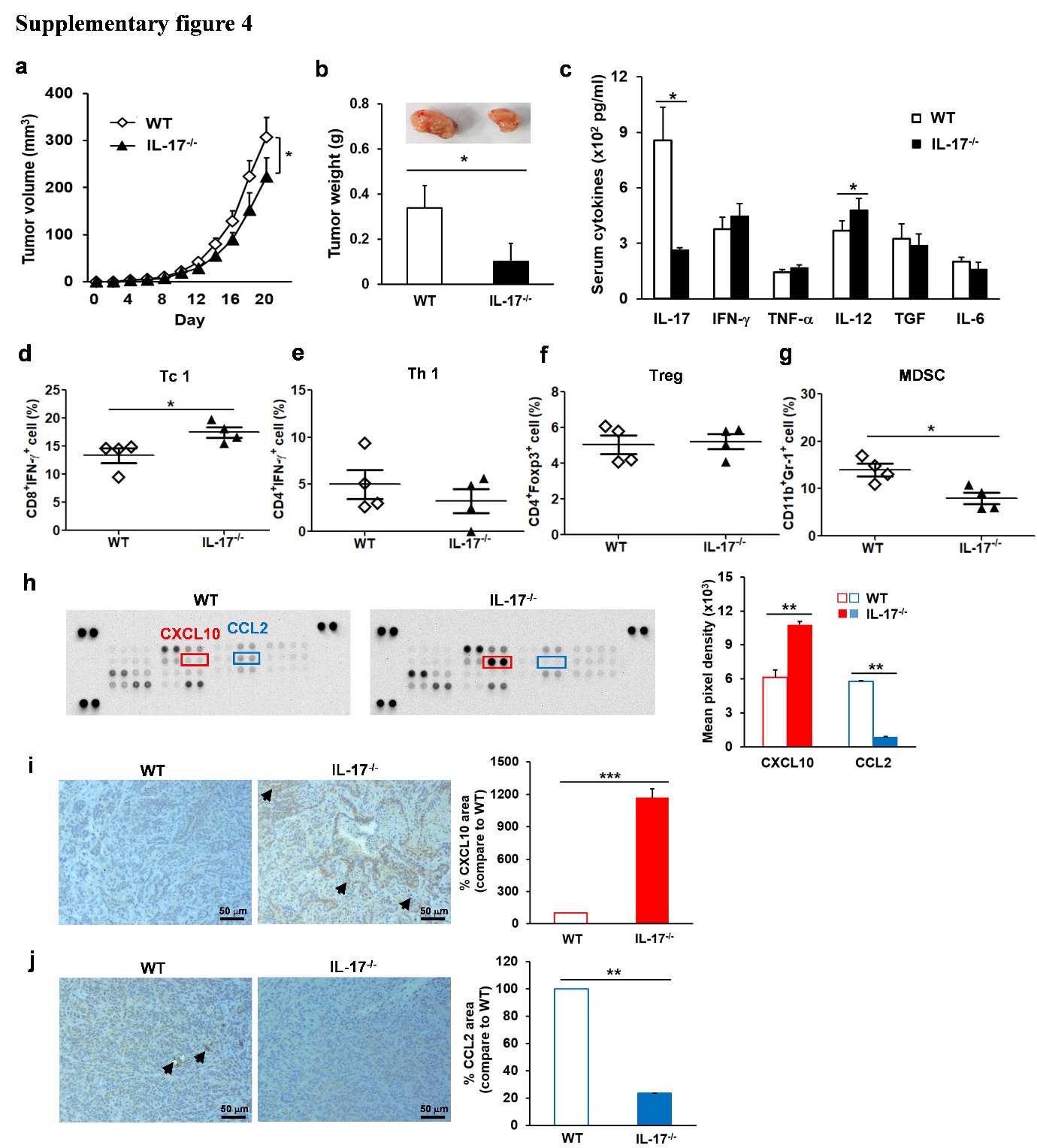
 Supplementary Figure S5. IL-17 deficiency attenuates tumor growth and enhances antitumor immunity in PDAC-bearing mice.** (a, b) Tumorigenesis in wild-type (WT) or IL-17^⁻/⁻^ mice implanted subcutaneously with Pan18 cells (N=4). Tumor volume was measured every two days (a), and tumor weight was recorded at endpoint (b). (c–g) Systemic and intratumoral immune responses in WT or IL-17^⁻/⁻^ PDAC-bearing mice (N=4). Serum IL-17, IFN-γ, TNF-α, IL-12, TGF-β, and IL-6 levels were quantified by ELISA (c). Frequencies of tumor-infiltrating Tc1 cells (d), Th1 cells (e), Treg cells (f), and MDSCs (g) were assessed by flow cytometry (N=4). (h–j) Tumor chemokine profiling. Chemokine array analysis of tumor lysates (h) and representative immunohistochemical staining with quantification of CXCL10 (i) and CCL2 (j) (N=4). Data are presented as mean ± SEM. *P < 0.05, **P < 0.01, ***P < 0.001.

**
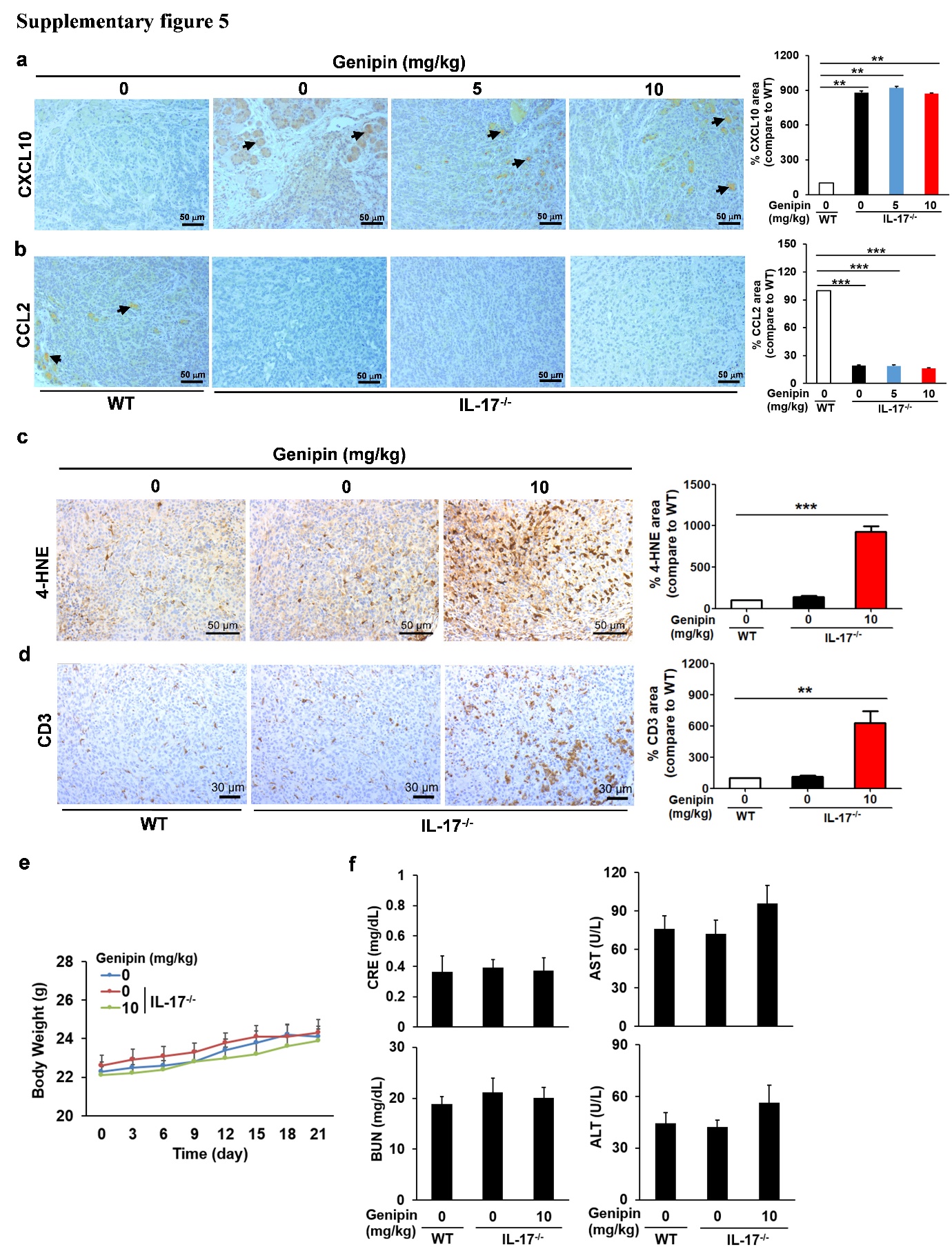
** **Supplementary Figure S6. Dual UCP2 and IL-17 blockade modulates chemokine expression, enhances lipid peroxidation and T-cell infiltration, and exhibits no systemic toxicity in PDAC-bearing mice** (see Figure 4a for experimental conditions). Representative immunohistochemistry and quantification of CXCL10 (a), CCL2 (b), 4-hydroxynonenal (4-HNE) (c), and CD3 (d) in tumor tissues from PDAC-bearing wild-type (WT) and IL-17^⁻/⁻^ mice treated with genipin (N = 4). Systemic tolerability of genipin treatment was assessed by monitoring body weight (e) and serum biochemical indicators of hepatic (AST, ALT) and renal (BUN, CRE) function (f) (N = 4–5). Data are presented as mean ± SEM. *P < 0.05, **P < 0.01, ***P < 0.001.

**
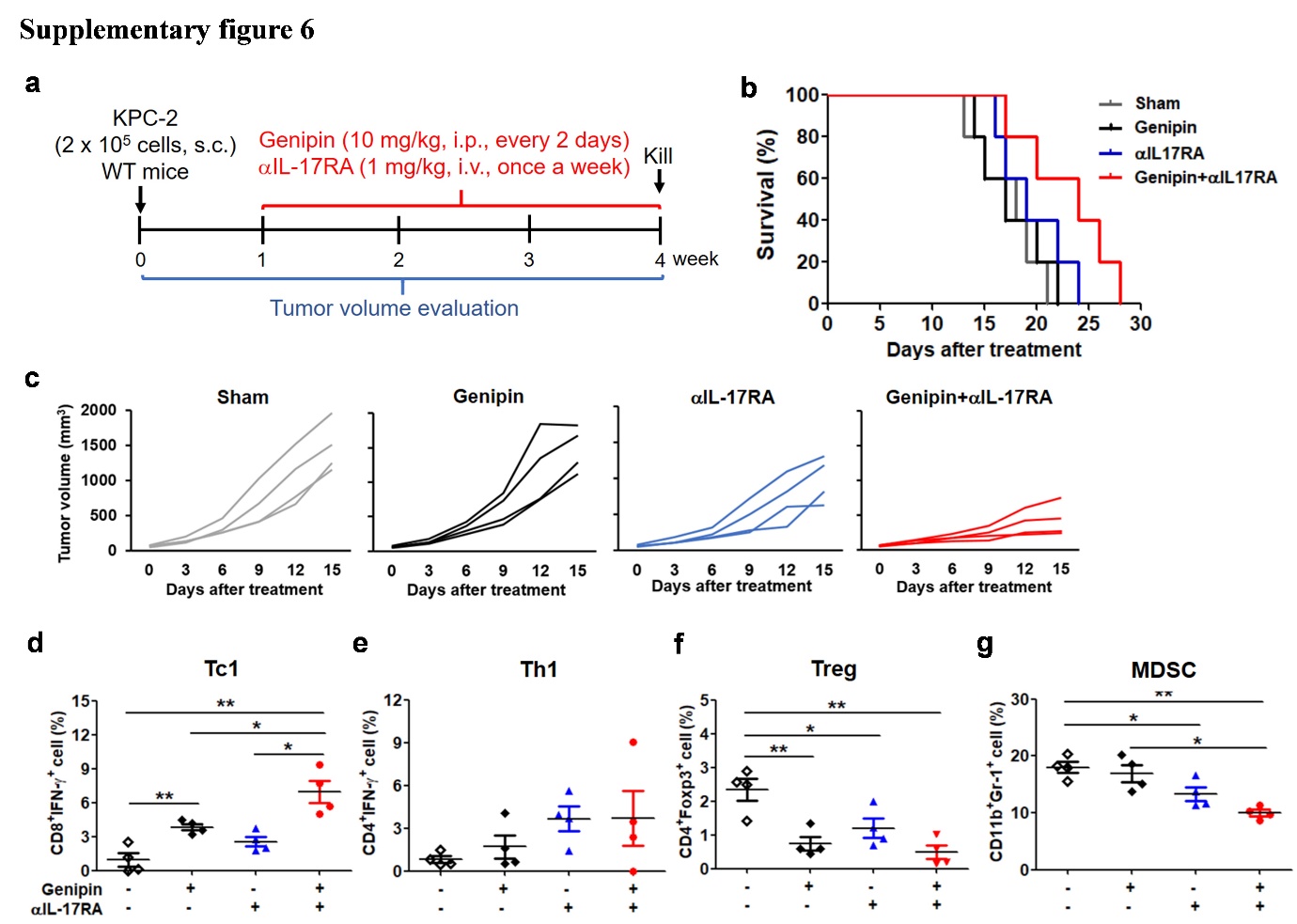
 Supplementary Figure S7. Combined UCP2 inhibition and IL-17RA blockade suppress tumor progression and enhance antitumor immunity in KPC-2–bearing mice.** (a–g) Experimental design, survival, tumor burden, and immune profiling in KPC-2–bearing wild-type mice treated with genipin and recombinant αIL-17RA. Mice received subcutaneous implantation of KPC-2 cells, followed by genipin (10 mg/kg, i.p., every two days) and αIL-17RA (1 mg/kg, i.v., once weekly) for 4 weeks. Study schema (a), overall survival (N = 5; b), tumor volume (N = 4; c), and intratumoral immune composition (N = 4; d–g) are shown. Frequencies of tumoral Tc1 (d), Th1 (e), Treg (f), and MDSC (g) populations were quantified by flow cytometry. Data represent mean ± SEM. *P < 0.05, **P < 0.01, ***P < 0.001.

**
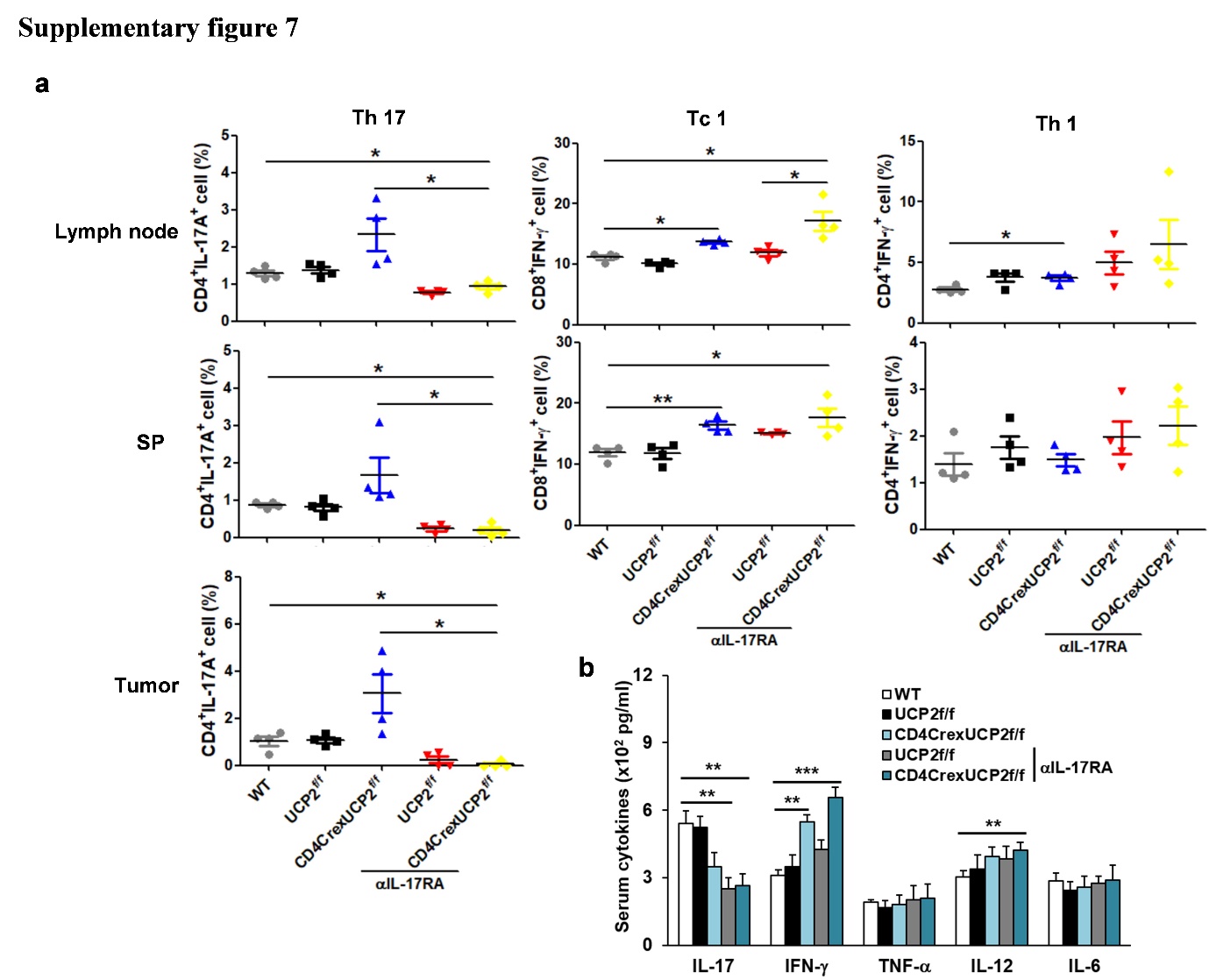
**

**Supplementary Figure S8.** **UCP2 deletion in CD4/CD8 T cells combined with αIL-17RA augments systemic and intratumoral immune activation in PDAC-bearing mice** (See Figure 6a, b for experimental design and treatment schema). (a) Flow-cytometric profiling of Tc1, Th1, and Th17 subsets in lymph nodes and spleens, and Th17 populations within tumor tissues from PDAC-bearing CD4Cre×UCP2^f/f^ mice treated with αIL-17RA. (b) Serum cytokine levels in PDAC-bearing CD4Cre×UCP2^f/f^ mice following αIL-17RA administration. Data represent mean ± SEM. *P < 0.05, **P < 0.01, ***P < 0.001.

**
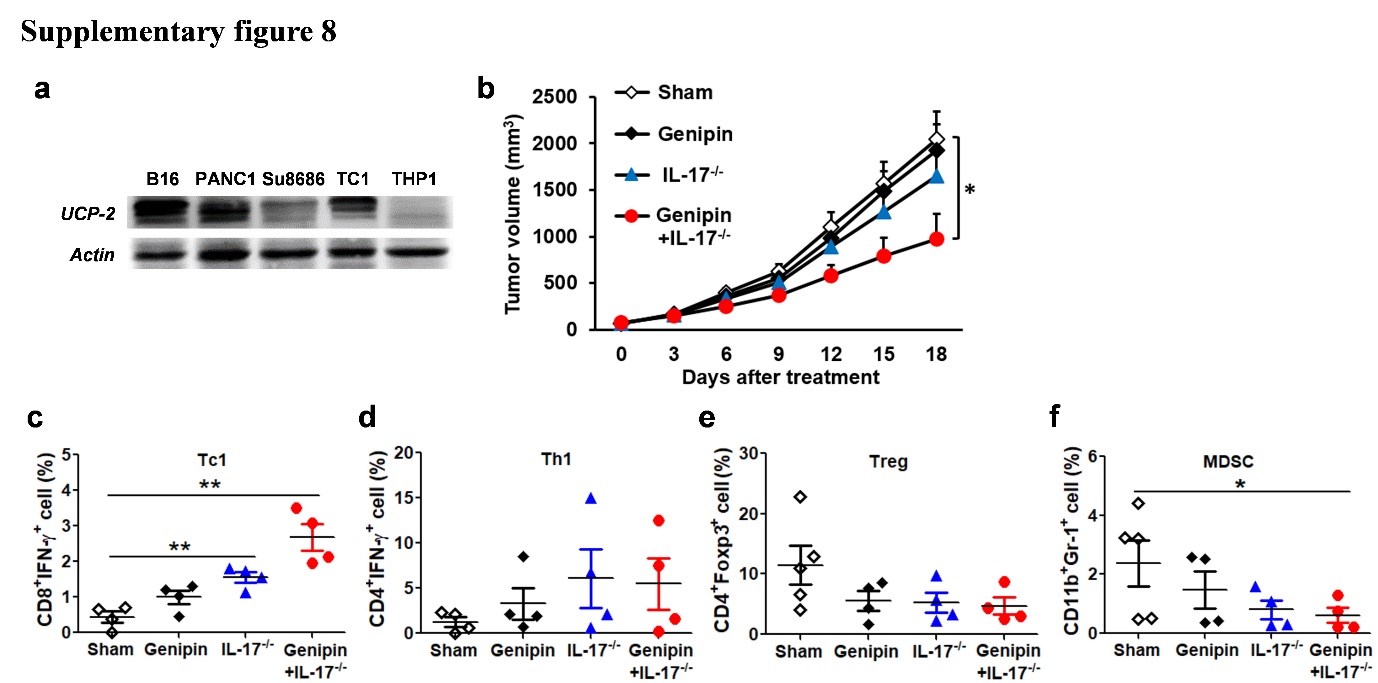
 Supplementary Figure S9. UCP2 inhibition combined with IL-17 depletion suppresses melanoma growth and enhances antitumor immunity.** (a) UCP2 expression across multiple cancer cell lines, including melanoma (B16), pancreatic cancer (PANC-1, SU.86.86), myeloma (TC-1), and leukemia (THP-1). (b-f) Tumor growth and immune profiling in melanoma-bearing WT or IL-17^⁻/⁻^ mice treated with genipin. Mice were implanted subcutaneously with B16F10 cells and administered genipin (10 mg/kg, i.p., every other day) for 3 weeks (N=4). Tumor growth curves (b) were recorded bi-daily. Flow-cytometric quantification of tumor-infiltrating Tc1 (c), Th1 (d), Treg (e), and MDSC (f) populations is shown. Data represent mean ± SEM. *P < 0.05, **P < 0.01, ***P < 0.001.

**Table S1. List of genes activating or inhibiting by genipin in Tc1.**

| **NO.** | **Gene** | **Description** |
| --- | --- | --- |
| 1 | Ctla2b | cytotoxic T lymphocyte-associated protein 2 beta |
| 2 | Slc35d3 | solute carrier family 35, member D3 |
| 3 | Pdia5 | protein disulfide isomerase associated 5 |
| 4 | Chit1 | chitinase 1 (chitotriosidase) |
| 5 | Wnt10b | wingless-type MMTV integration site family, member 10B |
| 6 | 1500009L16Rik | RIKEN cDNA 1500009L16 gene |
| 7 | Tpi1 | triosephosphate isomerase 1 |
| 8 | Prkcdbp | protein kinase C, delta binding protein |
| 9 | Gm14085 | predicted gene 14085 |
| 10 | Tbx21 | T-box 21 |
| 11 | Serpine2 | serine (or cysteine) peptidase inhibitor, clade E, member 2 |
| 12 | Cd79a | CD79A antigen (immunoglobulin-associated alpha) |
| 13 | Ifit1 | interferon-induced protein with tetratricopeptide repeats 1 |
| 14 | St6gal1 | beta galactoside alpha 2,6 sialyltransferase 1 |
| 15 | Acpp | acid phosphatase, prostate |
| 16 | Nipal1 | NIPA-like domain containing 1 |
| 17 | Serpinb6b | serine (or cysteine) peptidase inhibitor, clade B, member 6b |
| 18 | Spsb1 | splA/ryanodine receptor domain and SOCS box containing 1 |
| 19 | Bco2 | beta-carotene oxygenase 2 |
| 20 | Gm7334 | B-cell translocation gene 3 pseudogene |
| 21 | Fam101b | refilin B |
| 22 | Ifit3 | interferon-induced protein with tetratricopeptide repeats 3 |
| 23 | Pydc3 | interferon activated gene 208 |
| 24 | Serpinc1 | serine (or cysteine) peptidase inhibitor, clade C (antithrombin), member 1 |
| 25 | Ptgr1 | prostaglandin reductase 1 |
| 26 | Cd4 | CD4 antigen |
| 27 | Ptpn5 | protein tyrosine phosphatase, non-receptor type 5 |
| 28 | Nacc2 | nucleus accumbens associated 2, BEN and BTB (POZ) domain containing |
| 29 | Cdc42bpb | CDC42 binding protein kinase beta |
| 30 | Gls2 | glutaminase 2 (liver, mitochondrial) |
| 31 | Bspry | B-box and SPRY domain containing |
| 32 | Crispld2 | cysteine-rich secretory protein LCCL domain containing 2 |
| 33 | Pf4 | platelet factor 4 |
| 34 | Esm1 | endothelial cell-specific molecule 1 |
| 35 | Malat1 | metastasis associated lung adenocarcinoma transcript 1 (non-coding RNA) |
| 36 | Cd22 | CD22 antigen |
| 37 | Dnph1 | 2'-deoxynucleoside 5'-phosphate N-hydrolase 1 |
| 38 | Nt5dc2 | 5'-nucleotidase domain containing 2 |
| 39 | Rtp4 | receptor transporter protein 4 |
| 40 | Zbtb32 | zinc finger and BTB domain containing 32 |
| 41 | Neb | nebulin |
| 42 | Kcna2 | potassium voltage-gated channel, shaker-related subfamily, member 2 |
| 43 | Prdm1 | PR domain containing 1, with ZNF domain |
| 44 | Tbx21 | T-box 21 |
| 45 | Il12rb2 | interleukin 12 receptor, beta 2 |
| 46 | Il17a | interleukin 17A |
| 47 | Ccl5 | chemokine (C-C motif) ligand 5 |
| 48 | Ctla2a | cytotoxic T lymphocyte-associated protein 2 alpha |
| 49 | Ccl6 | chemokine (C-C motif) ligand 6 |
| 50 | Cd274 | CD274 antigen, PD-1 |
| 51 | Aldoc | aldolase C, fructose-bisphosphate |
| 52 | Pfkl | phosphofructokinase, liver, B-type |
| 53 | Pgam1 | phosphoglycerate mutase 1 |
| 54 | Ldha | lactate dehydrogenase A |
| 55 | Acat2 | acetyl-Coenzyme A acetyltransferase 2 |
| 56 | Eno2 | enolase 2, gamma neuronal |
| 57 | Hk2 | hexokinase 2 |
| 58 | Stat1 | signal transducer and activator of transcription 1 |
| 59 | Eomes | eomesodermin |
| 60 | Gck | glucokinase |

**Table S2. PDAC human subjects.**

| **Patient NO.** | **Gender** | **Age** | **AJCC stage** |
| --- | --- | --- | --- |
| 1 | Male | 56 | cT2NxM1, stage IV |
| 2 | Female | 57 | pT2N2M0, stage III |
| 3 | Male | 60 | cT3N0M1, stage IV |
| 4 | Female | 75 | cT4N0M0, stage III |
| 5 | Female | 47 | cT3N1M0,stage III |
| 6 | Female | 68 | cT4N1M1,stage IV |
| 7 | Male | 78 | - |
| 8 | Female | 74 | cT3N0M1, stage IV |
| 9 | Female | 52 | pT4N2cM0, stage III |
| 10 | Female | 77 | pT2N2cM0, stage III |
